# Spatial optimization of hierarchical healthcare facilities driven by multi-source data: a case study of Shenyang, China

**DOI:** 10.3389/fpubh.2025.1640070

**Published:** 2025-07-25

**Authors:** Yifei Wang, Duanqiang Zhai, Wuqi Xie, Shan Huang

**Affiliations:** ^1^College of Architecture and Urban Planning, Tongji University, Shanghai, China; ^2^Innovation and Research Center, Shanghai Tongji Urban Planning and Design Institute Co., Ltd., Shenyang, China; ^3^Shenyang Planning and Design Institute Co., Ltd., Shenyang, China; ^4^College of Architecture and Urban Planning, Shenyang Jianzhu University, Shenyang, China

**Keywords:** spatial equity, hierarchical healthcare facilities, multi-source data, service area analysis, accessibility optimization, location-allocation model

## Abstract

Amid rapid urbanization and accelerated population aging, spatial inequality in the distribution of healthcare facilities has become a pressing challenge in Shenyang. The dual problem of overconcentration of high-level medical resources in the urban core and insufficient primary care provision in peripheral areas highlights systemic imbalances in healthcare equity and efficiency. Grounded in the concept of spatial equity, this study integrates multi-source data—including population statistics, facility locations, and transportation networks—using advanced spatial analysis and big data fusion techniques. Through kernel density estimation, bivariate spatial autocorrelation, and service area network analysis, the spatial distribution and accessibility patterns of healthcare facilities across tertiary, secondary, and primary levels are comprehensively evaluated. To quantify spatial inequity, the Gini coefficient is introduced, confirming significant disparities in per capita healthcare resource allocation across administrative units. By combining service coverage modeling and the Location-Allocation (LA) model, the study identifies “healthcare deserts” and proposes a multi-tiered spatial optimization strategy aligned with China’s hierarchical diagnosis and treatment system. Simulation results demonstrate a pronounced “central concentration–peripheral scarcity” pattern, with particularly acute deficiencies in districts such as Shenbei and Hunnan. The planning intervention recommends the addition of six tertiary and six secondary/primary hospitals, along with the spatial reconfiguration of 260 community health service stations, increasing the overall population coverage rate to 98.98%. This research offers empirical evidence and a transferable planning framework for improving healthcare spatial equity through a “core decongestion–periphery reinforcement” approach. It also highlights the role of policy-guided developer participation and collaborative governance in enhancing service provision in newly urbanized areas. The study contributes practical insights for building an accessible, efficient, and resilient multi-level healthcare system, supporting the goals of the “Healthy Shenyang” initiative and offering a replicable model for similar urban contexts.

## 1 Introduction

Public service facilities are essential physical infrastructures for urban functioning and residents’ well-being, encompassing sectors such as healthcare, education, and finance (1). In the process of developing these areas and providing the corresponding facilities, developers have played a supporting role in urban development through policy incentives such as floor area ratio bonuses and mixed-use development plans (2, 3). Among these, healthcare facilities are of particular urgency and necessity due to their direct relationship with human health (4), which constitutes the fundamental premise for quality of life and societal development (5, 6). Equitable access to healthcare services lies at the heart of the public service system and serves as a crucial indicator of social equity and urban governance capacity (7, 8). With the rapid advancement of global urbanization and the intensifying issue of population aging, urban healthcare demand has been rising sharply, making the spatial distribution of healthcare resources a prominent challenge in large and medium-sized cities (9, 10). Healthcare systems often face problems such as resource scarcity, uneven distribution, and high access costs—becoming major bottlenecks in improving the quality and efficiency of public health services. According to the World Health Organization (WHO), high-quality healthcare services are not only critical to improving population health but also foundational to achieving social equity and inclusive development. The spatial allocation of healthcare facilities directly affects residents’ access to medical care and the fairness of resource distribution (11). As such, the spatial layout and accessibility of healthcare infrastructure have become key factors influencing the equity and efficiency of urban public health services.

In this context, countries around the world have increasingly emphasized the spatial planning of healthcare facilities. Developed nations such as the United Kingdom (12), the United States (13), New Zealand (14), Finland (15), and Germany (16) have established relatively systematic healthcare service systems and continue to advance spatial accessibility evaluations and optimization research. Even in developing countries like Brazil (17), Rwanda (18), and Malawi (19), relevant studies on the spatial equity of healthcare resources are gradually emerging despite limited infrastructure. Beyond spatial optimization, community participation is widely believed to be beneficial to the development, implementation and evaluation of health services (20). These global experiences consistently demonstrate that the spatial configuration of healthcare facilities is not merely a technical planning issue but also a critical topic concerning the delivery of basic public services, the promotion of social justice, and the realization of health equity.

As the world’s most populous developing country, China has long faced significant regional disparities in healthcare resource allocation and an unbalanced urban–rural distribution. In recent years, the national government has prioritized improving healthcare equity and service quality to narrow health service gaps among its 1.44 billion people (3, 21). Rapid urbanization has contributed to narrowing regional differences in per capita healthcare facility availability (22), yet spatial inequality has increasingly shifted from the rural–urban divide to intra-urban structural disparities (23), particularly within large metropolitan areas.

This persistent spatial mismatch stems not only from demographic and institutional factors but also from deep-rooted deficiencies in China’s traditional planning practices. Since the early stage of reform and opening-up, socioeconomic deregulation has spurred massive population flows into major urban centers, resulting in significant intra-city social stratification and spatial tension in public service provision (24). During this period, urban planning—especially residential neighborhood planning—largely relied on uniform “per capita facility indicators,” focusing on the quantity rather than the spatial suitability or community-specific needs of public services. This technocratic approach often ignored variations in residents’ health demands, mobility capacities, and neighborhood characteristics, resulting in spatial patterns that failed to ensure equitable access to healthcare (25). Furthermore, early healthcare infrastructure planning tended to emphasize coverage volume while overlooking the functional hierarchy and service stratification necessary for an efficient multi-tier healthcare system. Combined with socioeconomic disparities in income, education, and occupational status, these planning inadequacies have further reinforced unequal accessibility, particularly for vulnerable populations in peripheral or underserved urban districts (26).

Consequently, China presents a typical case of spatial imbalance in the allocation of public service resources, where ongoing reforms in healthcare delivery must be matched by spatial optimization strategies that better reflect demographic dynamics, behavioral patterns, and urban development trends (27).

Existing research on healthcare accessibility has made substantial progress, focusing primarily on three areas. First, spatial optimization of facility location includes both the restructuring of existing facilities (28, 29) and the rational allocation of newly added resources (30) to enhance overall resource utilization efficiency. Second, in terms of methodology, traditional approaches—such as shortest path analysis (31), kernel density estimation (32), and gravity models (33)—have been increasingly supplemented or replaced by more sophisticated models that integrate multi-source data and GIS-based spatial techniques (34, 35). With greater accessibility to population and spatial data (36–38), researchers have adopted advanced methods such as the two-step floating catchment area (2SFCA) method (39), the three-step floating catchment area (3SFCA) method (40), Web Mapping APIs, and improved potential models (41) to produce more refined accessibility evaluations. Third, equity in healthcare has emerged as a core concern, focusing on ensuring the rights of vulnerable groups to medical services and achieving equitable distribution under resource constraints (42–44). To quantitatively evaluate spatial equity, inequality indices such as the Gini coefficient have been increasingly applied, enabling more objective assessment of disparities between population distribution and healthcare resource allocation (45, 46).

Despite these advances, two major gaps remain in the literature. First, most studies focus on single-tier facility optimization, overlooking the hierarchical nature of the healthcare system (47, 48). This neglect has led to weak coordination among different facility levels and undermined the effectiveness of hierarchical medical treatment policies (49–51). Second, few studies incorporate dynamic regulatory mechanisms into the spatial configuration of multi-tiered healthcare facilities to ensure effective coordination and service integration. Under the ongoing strain of limited medical resources, how to achieve coordinated planning across different levels of healthcare services has become a critical issue in improving both the equity and efficiency of urban healthcare systems.

This study takes Shenyang as a representative case to investigate spatial optimization strategies for hierarchical healthcare facilities using multi-source data and GIS-based spatial analysis methods. The overarching goal is to improve the spatial equity and operational efficiency of healthcare systems under a multi-tier structure. Specifically, this research aims to: (1) identify spatial imbalances and underserved areas in facility distribution, especially in rapidly urbanizing peripheral districts; (2) develop optimization strategies for different facility levels to enhance spatial coordination under the hierarchical diagnosis and treatment system; and (3) propose a dynamic spatial allocation model that integrates both incremental expansion and reallocation of existing resources. By addressing these goals, the study not only contributes to filling theoretical gaps in multi-level healthcare facility planning—particularly in spatial modeling and demand-responsive regulation—but also provides practical guidance for equitable and adaptive urban healthcare planning. The analytical framework proceeds in three stages: spatial diagnosis of imbalance, efficiency evaluation via service coverage modeling, and strategy formulation through location-allocation simulation. The findings are expected to offer transferrable insights for other rapidly growing cities seeking to enhance the sustainability and inclusivity of their public health systems.

## 2 Materials and methods

### 2.1 Study area and research objects

Shenyang, the capital city of Liaoning Province, is a national historical and cultural city, a key international hub in Northeast Asia, and a major node within China’s integrated transportation network. As a strategic gateway connecting the Beijing-Tianjin-Hebei region to Northeast China, Shenyang plays a central role in regional economic and urban development. The study area for this research is focused on the central urban districts of Shenyang, encompassing the entire administrative territories of Heping, Huanggu, Shenhe, and Dadong Districts, as well as most parts of Shenbei New District, Hunnan District, Sujiatun District, Tiexi District, and Yuhong District.

The research targets healthcare facilities that provide clinical services and require spatial allocation based on population distribution. These facilities include both hospitals and community health service institutions, which serve key roles in disease treatment, health promotion, and basic public health service provision. Based on public data from the Shenyang Municipal Health Commission, the study area contains a total of 57 tertiary hospitals, 252 primary and secondary hospitals, and 266 community health service stations. In line with China’s hierarchical healthcare system and the “tiered diagnosis and treatment” model, this study categorizes healthcare facilities into three spatial levels (52): (1) City-level facilities represented by tertiary hospitals, which serve as high-capacity regional medical centers; (2) District-level (or sub-regional) facilities including both primary and secondary hospitals that offer general outpatient and inpatient services within specific urban subregions; (3) Community-level facilities, mainly consisting of community health service stations, which provide essential public health and primary care services at the neighborhood scale.

Given the significant differences in service capacity—such as bed numbers, physician counts, and medical departments—between tertiary hospitals and lower-tier institutions, and the relatively similar average scale indicators between primary and secondary hospitals, this study groups primary and secondary hospitals into a single category for analytical purposes. This classification enables a three-tiered spatial analysis of healthcare accessibility and service allocation, supporting a differentiated yet integrated optimization strategy across levels.

### 2.2 Data sources

The data used in this study are diverse and encompass multiple aspects, including healthcare facilities, residential areas, road networks, and population distribution. To ensure accuracy and usability, all datasets underwent rigorous selection and preprocessing procedures. The sources and handling methods of the data are as follows:

(1) Population Distribution Data: The spatial population data were obtained from the Oak Ridge National Laboratory (ORNL) of the US Department of Energy. These datasets have a spatial resolution of 1 km and have been published annually since 2000. The original data were preprocessed in ArcGIS, including coordinate system conversion and format adjustments, to ensure compatibility with other spatial datasets. The processed data provide a reliable foundation for spatial analysis by accurately reflecting the residential distribution and supporting the integration with healthcare facility data for spatial optimization. In addition, the administrative unit-level population data of each subdistrict in Shenyang’s central urban area were primarily sourced from China’s Seventh National Population Census, ensuring the accuracy and authority of population statistics at the neighborhood scale.
(2) Healthcare Facility Data: The distribution of healthcare facilities was derived from the Point-of-Interest (POI) data of Baidu Maps. A custom Python crawler was used to extract relevant information, which was then cross-validated and categorized using publicly available data from the Shenyang Municipal Health Commission. Healthcare facilities were classified into four categories: tertiary hospitals, secondary hospitals, primary hospitals, and community health service stations. The classification was based on facility level and service capacity to ensure an accurate representation of the spatial layout and service functions of healthcare institutions in Shenyang.
(3) Residential Area Vector Data: Residential area data were also collected from Baidu Maps using Area-of-Interest (AOI) datasets. The “Residential Compound” layer was extracted using a Python crawler, providing vector location and total household information for each residential area in Shenyang. In this study, vacancy rates were not considered, and all recorded households were assumed to be occupied. This dataset serves as a basis for estimating healthcare service demand at the residential level and supports spatial analyses of service coverage.
(4) Road Network Data: Road network data were sourced from OpenStreetMap (OSM), containing vector information on road alignment, classification, length, and width. ArcGIS tools were employed to extract road centerlines, followed by classification based on standard urban road hierarchy: expressways, primary roads, secondary roads, and local roads. To further analyze accessibility, average travel speeds were assigned as follows: 80 km/h for expressways, 60 km/h for primary roads, 40 km/h for secondary roads, and 30 km/h for local roads. These data support the modeling of healthcare accessibility within complex urban transportation systems, enabling a refined evaluation of service capabilities across different facility levels.

### 2.3 Methods

#### 2.3.1 Gini coefficient

To quantify spatial equity in the distribution of healthcare facilities, this study applies the Gini coefficient, a well-established metric for measuring inequality, increasingly used in public service allocation studies. The coefficient is derived from the Lorenz curve and is calculated using the following formula:


$$G=1-\sum_{i=1}^{n}(P_{i}-P_{i-1})(R_{i}+R_{i-1})$$

where $P_{i}$ represents the cumulative proportion of the population and $R_{i}$ the cumulative proportion of healthcare resources (measured by hospital bed counts) for the i th spatial unit, and n is the total number of sub-districts.

In this study, population data at the sub-district level were obtained from the **S**eventh National Population Census (2020) for central Shenyang. The number of hospital beds from primary, secondary, and tertiary facilities within each sub-district was used to represent healthcare capacity. By mapping the distribution of population and resources, we calculated the Gini coefficient to assess spatial equity.

#### 2.3.2 Spatial distribution analysis of healthcare facilities in Shenyang

(1) Kernel Density Estimation (KDE): Kernel density estimation is a widely used spatial analysis technique for evaluating the distribution patterns of healthcare facilities. By applying KDE to different levels of medical institutions, this study identifies areas of high and low facility density within the urban region. The estimation is expressed as follows:

$$D(x,y)=\frac{1}{h^{2}}\sum_{i=1}^{N}K(\frac{d((x,y),(x_{i},y_{i}))}{h})$$

Where D(x,y) is the estimated density at location (x,y) , N is the total number of facility points, K is the kernel function, h is the bandwidth (smoothing parameter), and $\left(x_{i},y_{i}\right)$ denotes the coordinates of the i th facility. This method enables identification of over-concentrated and underserved areas across Shenyang’s urban landscape.

(2) Standard Deviation Ellipse Analysis: This method is employed to measure the directional trends and spatial spread of healthcare facilities at various levels (e.g., tertiary, secondary). By calculating the geometric center, orientation, and dispersion of facility distributions, standard deviation ellipses provide insight into spatial aggregation tendencies. The ellipses are defined by:

$$\sigma_{x},\sigma_{y},\theta$$

Where $\sigma_{x}$ and $\sigma_{y}$ are the standard deviations along the x and y axes, and θ is the rotation angle representing the major axis direction. This analysis helps compare spatial characteristics and imbalances among different levels of facilities.

(3) Bivariate Spatial Autocorrelation: This technique investigates the spatial relationship between healthcare facilities and population distribution to uncover mismatches in supply and demand. Using GeoDa software, bivariate Moran’s I is calculated as:

$$I=\frac{N\sum_{i}\sum_{j}w_{\mathrm{ij}}(X_{i}-X)(Y_{j}-\bar{Y}))}{\sum_{i}{\left(X_{i}-\bar{X}\right)}^{2}\sum_{j}{\left(Y_{j}-\bar{Y}\right)}^{2}}$$

Where N is the number of spatial units, $w_{\mathrm{ij}}$ represents the spatial weight between areas i and j , and X , Y represent attribute values for facilities and population, respectively. A significant deviation of Moran’s I from zero indicates spatial correlation, revealing potential mismatches between healthcare supply and residential demand.

#### 2.3.3 Service area analysis

Service area analysis, based on road network data, is used to calculate the spatial coverage of healthcare facilities. Utilizing the network analysis module in ArcGIS and integrating road vector data, the analysis determines the service radius of each facility and identifies underserved or “medical desert” zones. The service area $S_{i}$ of facility ii is defined as:


$$S_{i}=(j\;|\;d_{\mathrm{ij}}\leq R_{i})$$

Where $d_{\mathrm{ij}}$ is the network distance from facility i to residential area j , and $R_{i}$ is the maximum service radius of facility i . This method identifies areas lacking adequate access to healthcare, providing critical input for subsequent spatial optimization.

#### 2.3.4 Location allocation model

The Location Allocation (LA) model is a classical optimization technique used to determine optimal facility locations based on spatial demand. The model aims to achieve the most efficient match between supply points (healthcare facilities) and demand points (residential areas), often optimizing for minimal distance or maximal service coverage. The general form of the objective function is:


$$\min\sum_{i=1}^{N}\sum_{j=1}^{M}c_{\mathrm{ij}}x_{j}$$

Subject to: $x_{j}\in (0,1):$ whether facility j is selected; $c_{\mathrm{ij}}$ : cost (distance or time) from demand point i to facility j ; $d_{\mathrm{ij}}$ : distance between i and j ; By adjusting the objective and constraints, the model can simulate various scenarios such as minimizing travel time, reducing service costs, or maximizing population coverage, thereby supporting optimal multi-level healthcare facility planning.

## 3 Results

### 3.1 Spatial distribution characteristics of population and healthcare facilities in Shenyang

#### 3.1.1 Healthcare service demand pattern: population structure and spatial distribution

**Figure 1:**
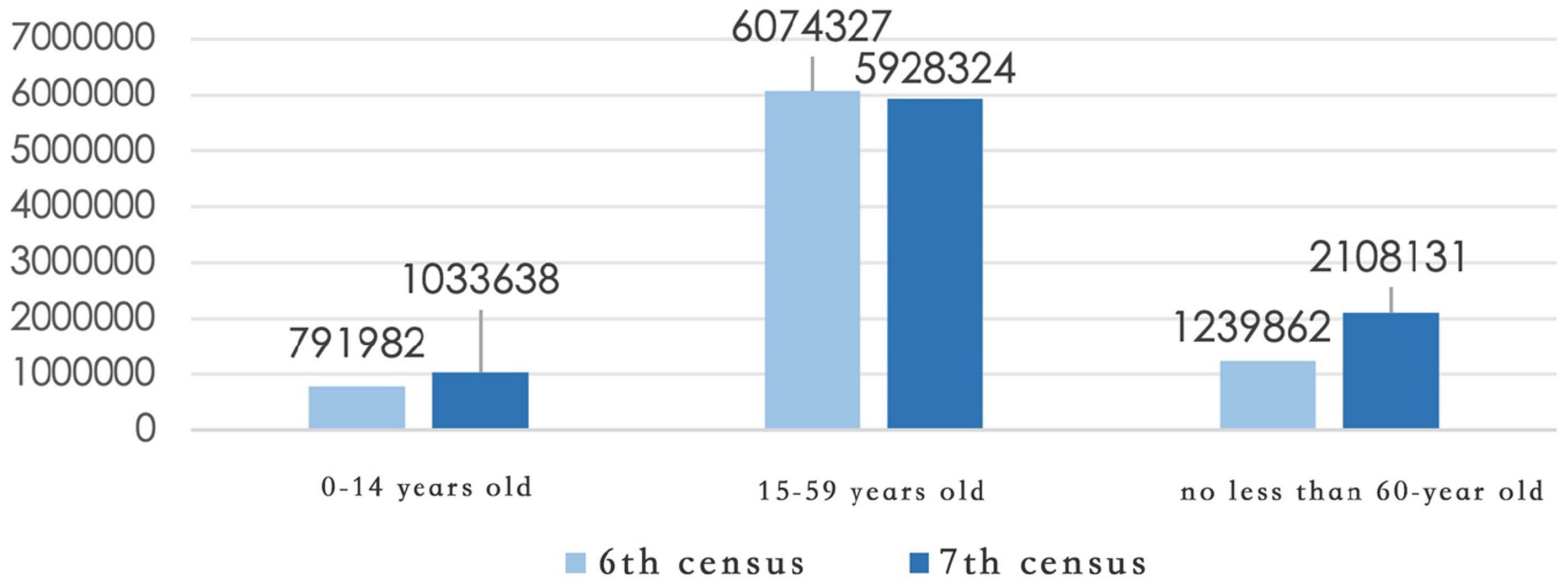
Population census data of Shenyang.

Before optimizing the spatial allocation of healthcare facilities, it is essential to comprehensively assess the urban population structure and healthcare service demand. According to the Seventh National Population Census (Figure 1), Shenyang’s permanent population includes 1,033,638 individuals aged 0–14 (11.40%), 5,928,324 individuals aged 15–59 (65.36%), and 2,108,131 individuals aged 60 and above (23.24%), of whom 1,403,246 (15.47%) are aged 65 and over. Compared with data from the Sixth Census in 2010, the aging trend in Shenyang has intensified significantly, with the proportion of people aged 60 and above increasing by 7.94 percentage points. Simultaneously, the share of children has also shown a slight increase. These trends indicate growing healthcare service needs from both ends of the age spectrum—the older adults and children—posing new challenges for future resource planning.

**Figure 2:**
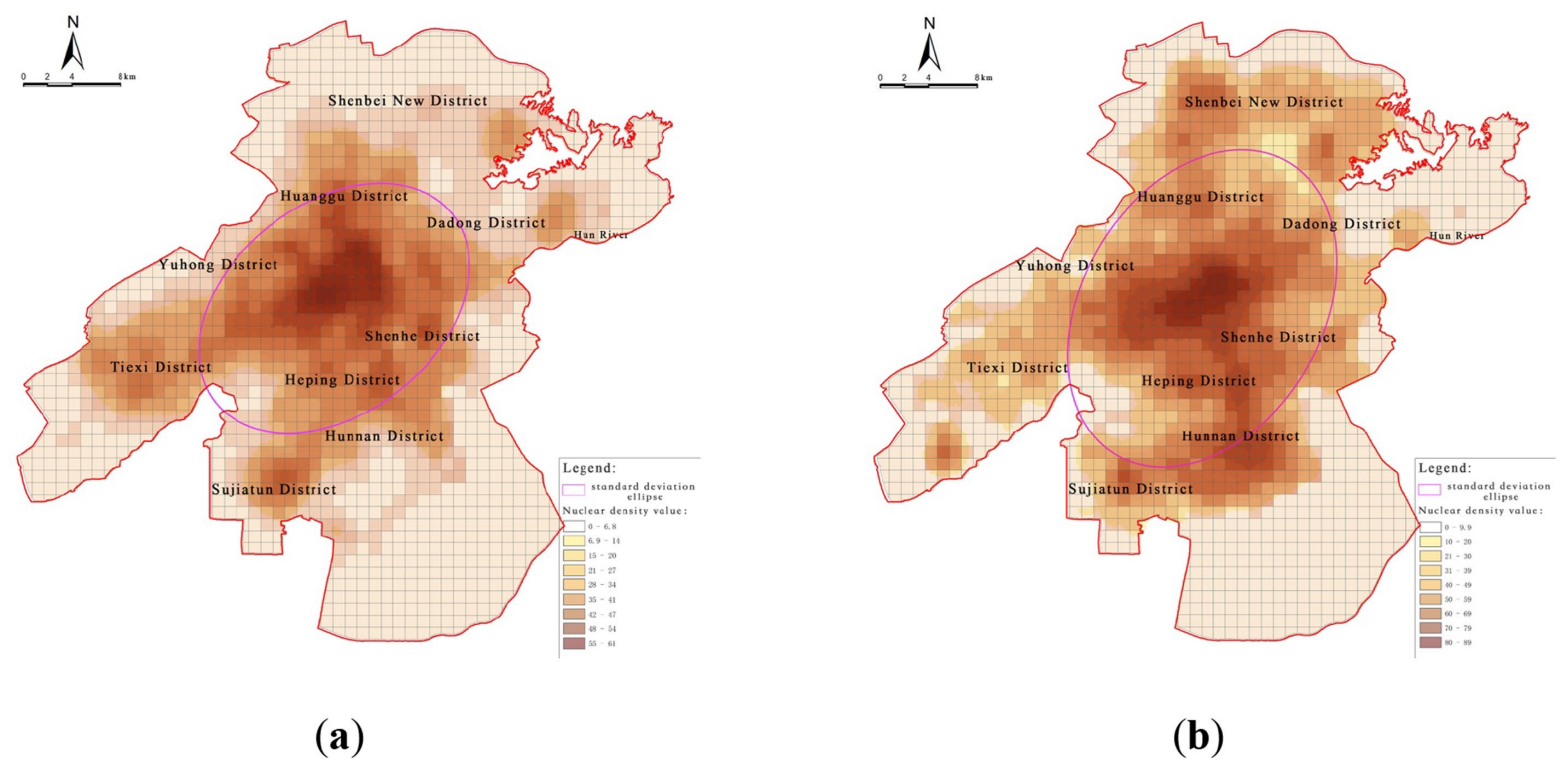
Spatial trends of population change. **(a)** Population distribution in central urban areas (2010); **(b)** Population distribution in central urban areas (2020).

In terms of spatial distribution, population centroid tracking from 2010 to 2020 using ArcGIS (Figure 2) shows a clear shift: while the population was historically concentrated in the old urban districts north of the Hun River, rapid urban expansion in the Hunnan District has resulted in the emergence of a new demographic center. However, this shift has not been met with a corresponding increase in healthcare infrastructure, revealing a structural mismatch between population migration and facility provision—what can be termed a “population shift–infrastructure lag” dilemma.

**Figure 3:**
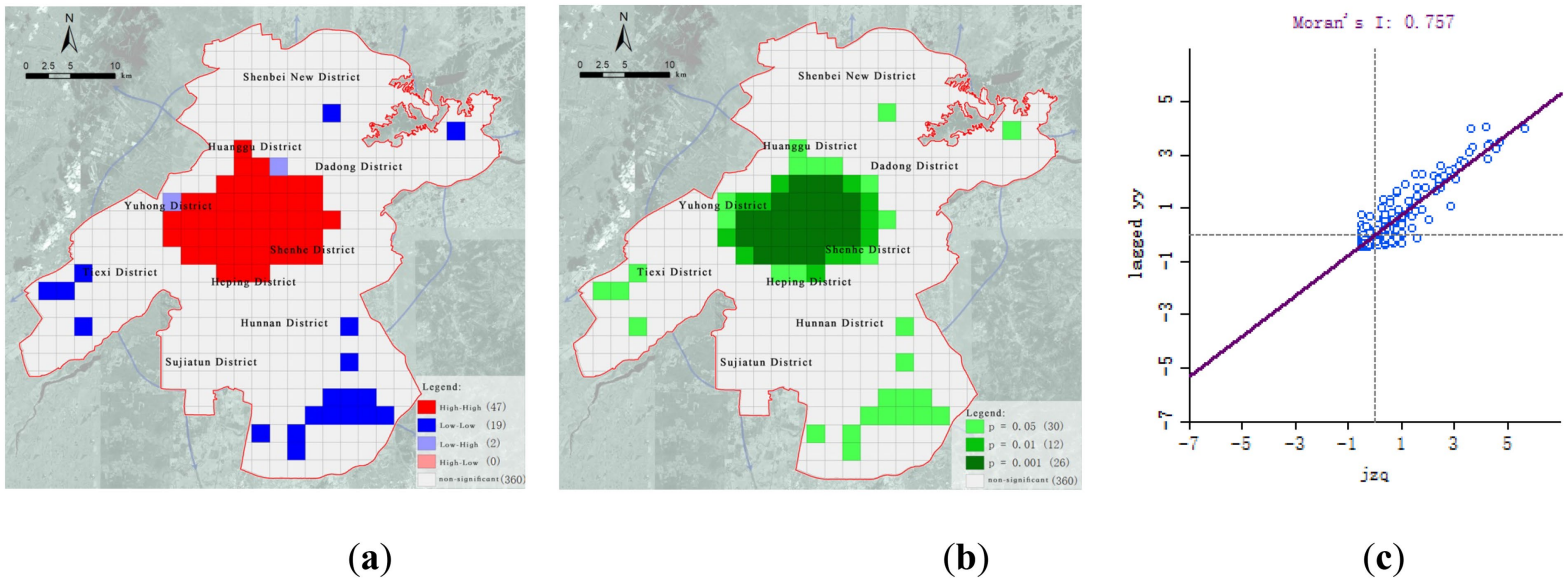
Bivariate spatial autocorrelation analysis of population and medical facilities. **(a)** High-low cluster map; **(b)** Significance map; **(c)** Moran scatterplot.

To further analyze this mismatch, a bivariate spatial autocorrelation analysis using GeoDa was conducted between population distribution and healthcare facility locations (Figure 3). The results reveal a significant spatial correlation across the entire study area. “High–high” clusters—areas with both high population density and high healthcare facility density—are primarily located in central urban districts such as Heping, Shenhe, and Tiexi, reflecting a strong coupling between service provision and demand. In contrast, “low–low” clusters—areas with both sparse population and inadequate healthcare facilities—are predominantly found on the urban periphery, including Shenbei New District and Sujiatun District, highlighting areas of dual deficiency.

Of particular concern is the Hunnan District, which, despite having developed into a new population hotspot, still lacks sufficient healthcare infrastructure. This region is at risk of becoming a future “medical service gap” zone unless timely interventions are made.

#### 3.1.2 Spatial distribution of city-level healthcare facilities

**Figure 4:**
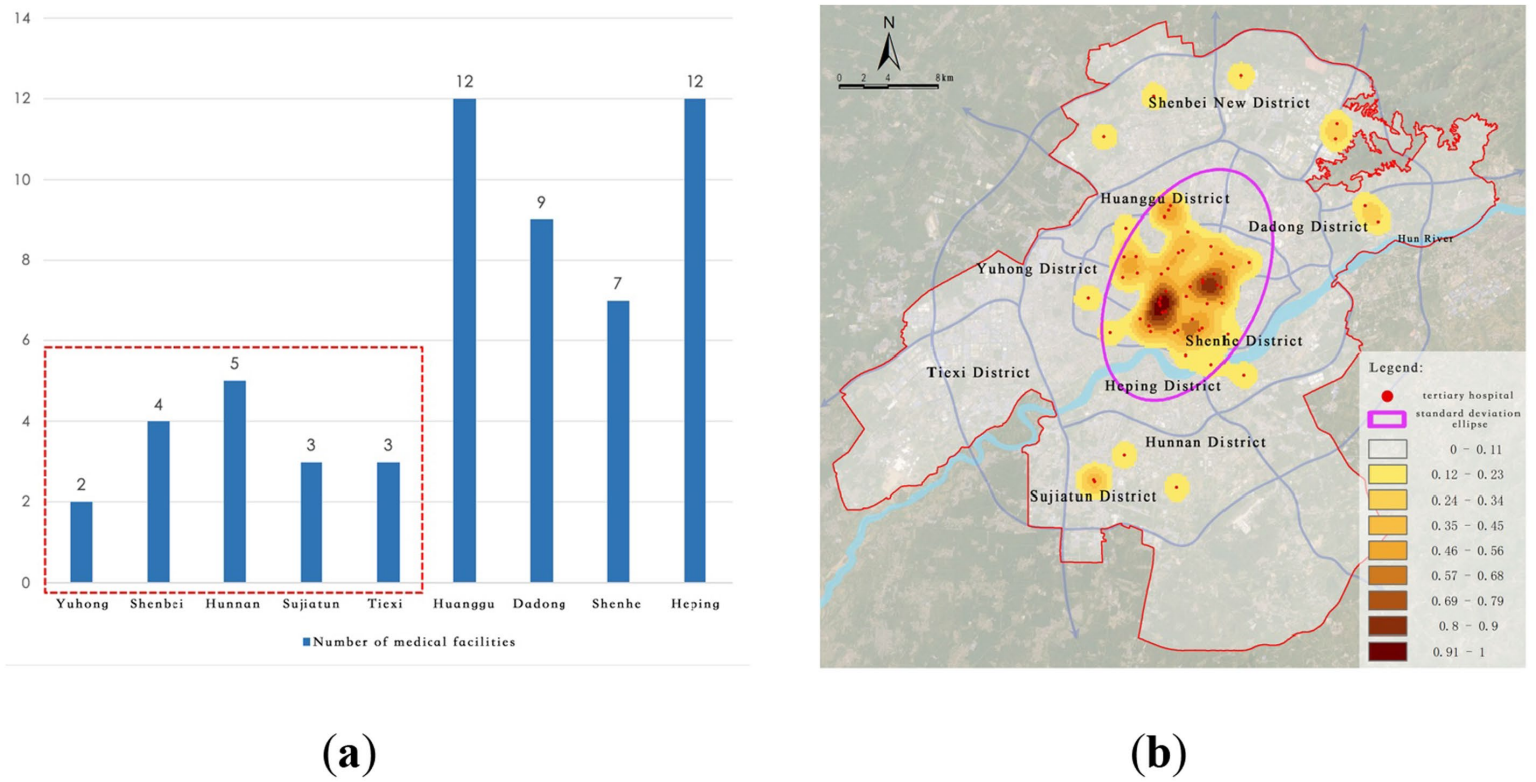
Current distribution of city-level medical facilities. **(a)** Number of tertiary hospitals; **(b)** Kernel density of tertiary hospitals.

City-level healthcare facilities primarily focus on treating complex and severe diseases, as well as providing high-end medical services. There are a total of 57 such facilities, mostly tertiary hospitals, exhibiting a “dual-core + clustered” centralized layout pattern (Figure 4). The core nodes are the First Affiliated Hospital of China Medical University and Shenyang First People’s Hospital, both located in Shenyang’s traditional central urban area. The spatial characteristics are as follows:

(1) High concentration at the center, insufficient distribution at the periphery: These facilities are mainly concentrated in the core urban areas, such as Heping District and Shenhe District, forming dense clusters of medical services. In contrast, peripheral areas like Shenbei New District, Yuhong District, and Sujiatun District are severely underserved, with significant service gaps in the southeastern regions.
(2) High density north of Hun River, service gaps south of Hun River: More than 80% of the city-level facilities are located in the old urban area north of the Hun River. However, the area south of the river, which serves as the city’s expansion axis, is notably lacking in high-level healthcare resources, unable to meet the increasing demand for cross-regional high-end medical services.

#### 3.1.3 Spatial distribution of district-level healthcare facilities

**Figure 5:**
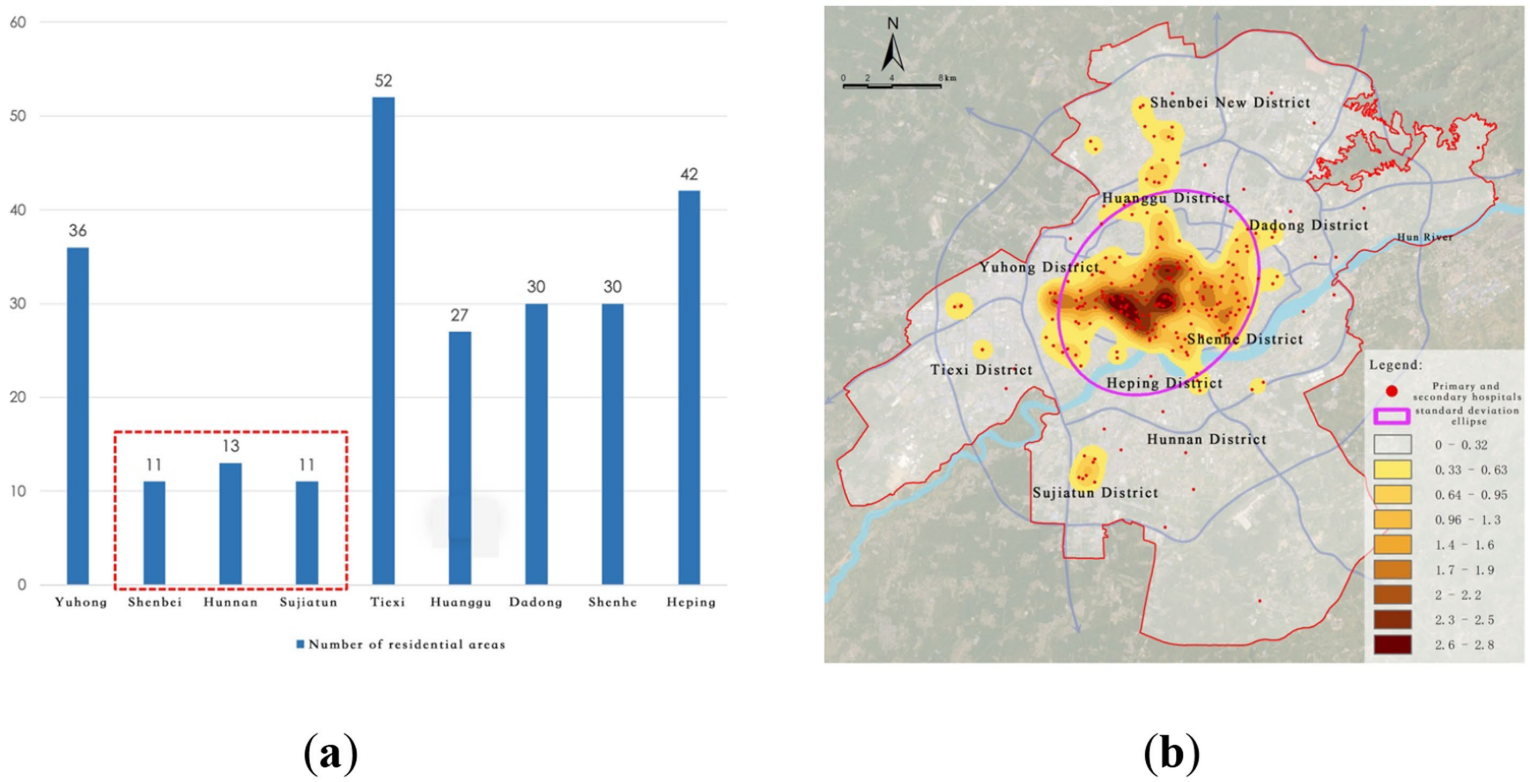
Current distribution of district-level medical facilities. **(a)** Number of primary and secondary hospitals; **(b)** Kernel density of primary and secondary hospitals.

District-level healthcare facilities primarily serve routine medical needs, consisting mainly of secondary and primary hospitals. A total of 252 such facilities are distributed in a relatively multi-center and widely covered pattern with a cluster + networked distribution (Figure 5). Core nodes include Tiexi Square, the First Affiliated Hospital of China Medical University, and Shenyang Fourth People’s Hospital, forming multiple district healthcare hubs. The spatial distribution exhibits the following characteristics:

(1) Broader coverage than city-level facilities, but significant structural disparities: Standard deviation ellipse analysis shows that the district-level facilities have a much wider coverage area compared to city-level facilities, covering much of the central and some sub-center regions. However, there are still notable service gaps or shortages in regions such as Shenbei New District, Hunnan District, and Sujiatun District.
(2) Insufficient service capacity in southern new towns: Although some facilities have expanded southward, primary and secondary hospitals in the southern area of the Hun River (especially in Hunnan New City) remain scarce. This misalignment with population growth impacts accessibility and service quality for routine medical care at the district level.

#### 3.1.4 Spatial distribution of community health service stations

**Figure 6:**
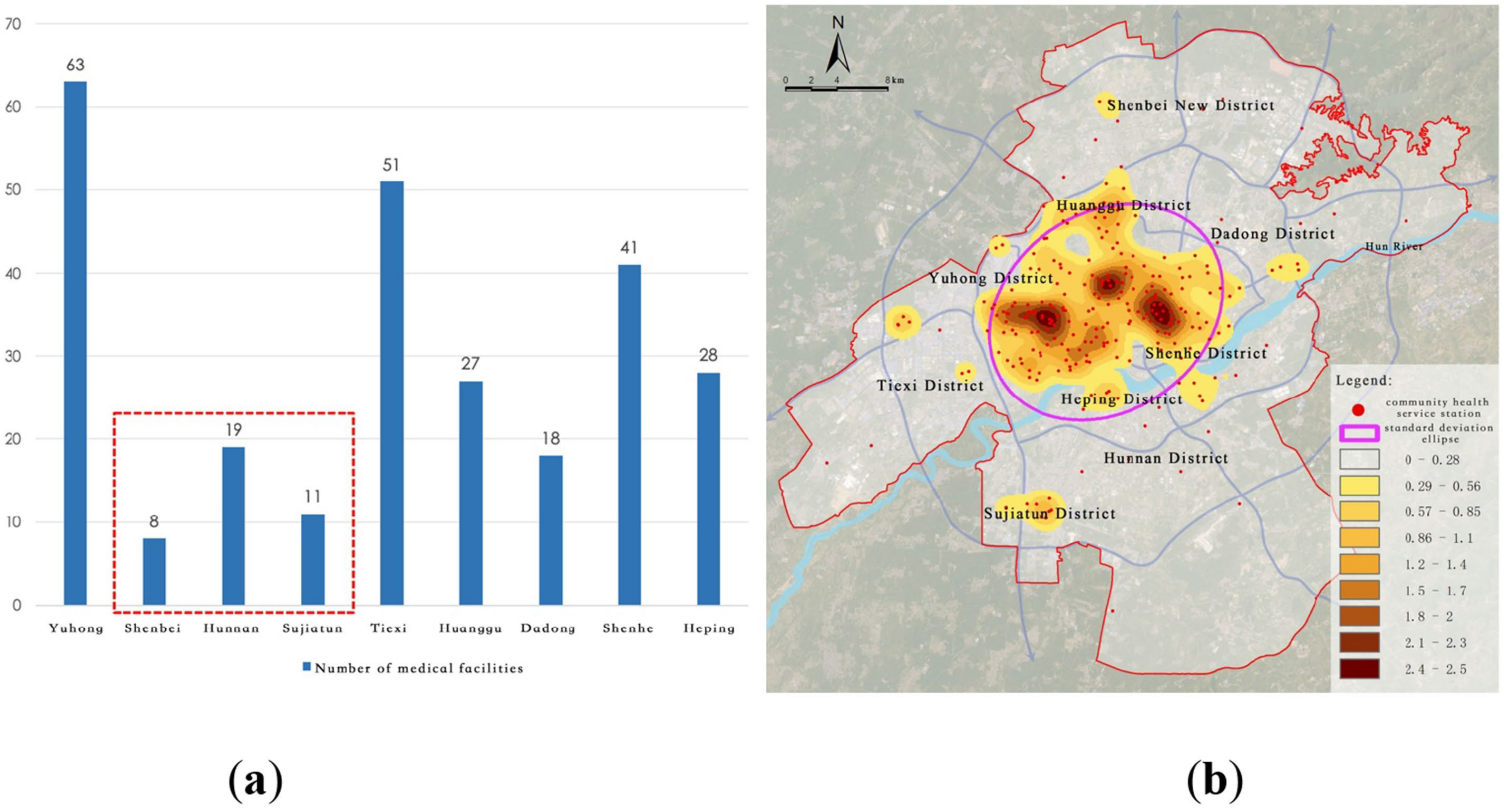
Current distribution of community-level medical facilities. **(a)** Number of community health service stations; **(b)**. Kernel density of community health service stations.

Community-level healthcare facilities form the backbone of the urban grassroots medical network, primarily consisting of 266 community health service stations. The distribution overall shows a typical decentralized + uneven pattern, with “dense in the old city, sparse in new districts” characteristics. In some areas, there is also a multi-center clustering phenomenon, with representative nodes including Tiexi Square, Kejian Park, and Huaiyuan Gate (Figure 6). The spatial characteristics are as follows:

(1) Severe imbalance in grassroots service resources: Community health service stations are predominantly located in the old urban areas, especially in historical districts such as Heping and Tiexi. In contrast, emerging development areas like Shenbei New District and Sujiatun District exhibit a significant shortage of facilities, leading to a weak service system.
(2) Severe lag in grassroots facilities in new towns: Particularly in the areas south of the Hun River, community health service stations are far fewer than those north of the river, with insufficient spatial coverage. This results in difficulties for residents seeking primary care for minor illnesses, which may drive patients to seek services from higher-level hospitals, creating resource misallocation and exacerbating diagnostic burdens.

#### 3.1.5 Spatial equity assessment based on Gini coefficient

The results show that the overall Gini coefficient for inpatient bed distribution in Shenyang’s central urban area is 0.523, significantly exceeding the internationally recognized alert threshold of 0.4. This indicates a marked spatial inequality in the allocation of healthcare resources relative to population distribution. Among districts (Table 1), Sujiatun District (0.68) and Hunnan District (0.62) exhibit the most severe disparities, where large proportions of the population reside in subdistricts with no hospital beds. In Sujiatun, for example, 5 out of 7 subdistricts—accounting for over 70% of the district population—lack any inpatient medical capacity. In contrast, Yuhong (0.31) and Huanggu (0.38) districts demonstrate relatively balanced distributions.

**Table 1:** Gini coefficients of medical resource distribution by district (based on street-level data of Shenyang central urban area).

District	Sub-districts	Population	Total beds	Gini coefficient	Inequality assessment
Sujiatun	7	242,680	225	0.683	Highly Unequal
Hunnan	11	1,069,857	4,285	0.615	Highly Unequal
Tiexi	14	1,426,170	4,840	0.484	Relatively Large Gap
Heping	10	699,224	6,610	0.575	Highly Unequal
Yuhong	9	1,013,438	4,075	0.312	Relatively Reasonable
Shenhe	12	1,021,345	5,750	0.492	Relatively Large Gap
Dadong	9	694,283	5,850	0.408	Relatively Large Gap
Huanggu	10	1,048,602	5,950	0.382	Relatively Reasonable
Shenbei	5	309,991	3,140	0.505	Highly Unequal

The analysis also reveals extreme internal disparities at the subdistrict level. For instance, Nanhudajie Subdistrict in Heping District, with a population of 102,608, concentrates 33% of the district’s total bed capacity (2,210 beds), while Wusan Subdistrict in Hunnan, home to over 223,000 residents, accounts for 36% of the district’s beds (1,535 beds). These findings highlight the mismatch between facility provision and population demand, particularly in newly developed residential clusters and urban fringe zones. This evidence further substantiates the spatial polarization of healthcare resources and underscores the need for targeted redistribution strategies in urban healthcare planning.

#### 3.1.6 Summary of the spatial distribution of tertiary healthcare facilities

Through the analysis of the spatial distribution of healthcare facilities and population, the following issues regarding Shenyang’s healthcare facility distribution have been identified:

(1) Increasing Demand for Medical Resources: On the one hand, as the core city of the Shenyang Metropolitan Area, Shenyang’s influence over surrounding cities is growing. This will likely lead to an increase in the number of patients traveling to Shenyang for medical treatment, which will result in both internal and external demand for medical resources. On the other hand, with the improvement in the economic and social development levels, citizens are expecting higher-quality and higher-level healthcare services.
(2) Changes in the Structure of Medical Service Demand: Shenyang has a large aging population, and the proportion of older adult individuals is gradually increasing. In addition, the introduction of policies encouraging multiple births has led to a higher birth rate. The healthcare demands for both the older adults and children are steadily growing, and in the future, the demand for medical resources will continue to rise.
(3) Need for Optimizing Medical Resource Allocation: The distribution of medical resources in the city is imbalanced. Tertiary hospitals are predominantly located in the old urban areas, leading to a concentration of highly educated and skilled healthcare professionals in these regions. This imbalance results in uneven development between different regions and institutions, and the healthcare market in the surrounding new districts still needs to be developed.
(4) Underdeveloped Grassroots Healthcare System: Community health service stations, as grassroots medical facilities, are often the first choice for residents seeking routine medical care. However, the uneven distribution of community health service stations makes it likely that residents with minor illnesses will seek treatment at larger hospitals, thus occupying limited resources at higher-level facilities. The spatial layout of grassroots healthcare facilities still needs further improvement.

### 3.2 Identification and judgment of “healthcare deficiency areas” in Shenyang

In determining the service radius parameters for healthcare facilities at different levels, this study refers to national regulatory documents such as the Healthcare Institution Establishment Plan and the Implementation Rules of the Healthcare Institution Administration Regulation, as well as relevant academic literature. It is generally recognized that the maximum acceptable travel time for vulnerable populations, such as children and the older adults, to access healthcare services is approximately 1.5 h (53, 54). Taking into account the average walking speed of these groups, the service radii were set as follows: 5 kilometers for tertiary hospitals, 3 kilometers for secondary hospitals, and 1 kilometer for community health service stations. The community-level thresholds were further refined into 1,000 meters, 500 meters, and 300 meters in accordance with the Standards for the 15-Minute Community Life Circle and the Urban Residential Area Planning and Design Standard. These parameters aim to balance the mobility characteristics of different population groups with the principle of spatial equity in healthcare access.

#### 3.2.1 “Healthcare deficiency” areas at the citywide level

Citywide medical facilities primarily include tertiary hospitals, which serve as the core of healthcare resources. Due to their advanced medical technology and extensive service coverage, the service radius of tertiary hospitals is set at 5,000 meters in this study to represent their service capability and coverage across the entire city.

**Figure 7:**
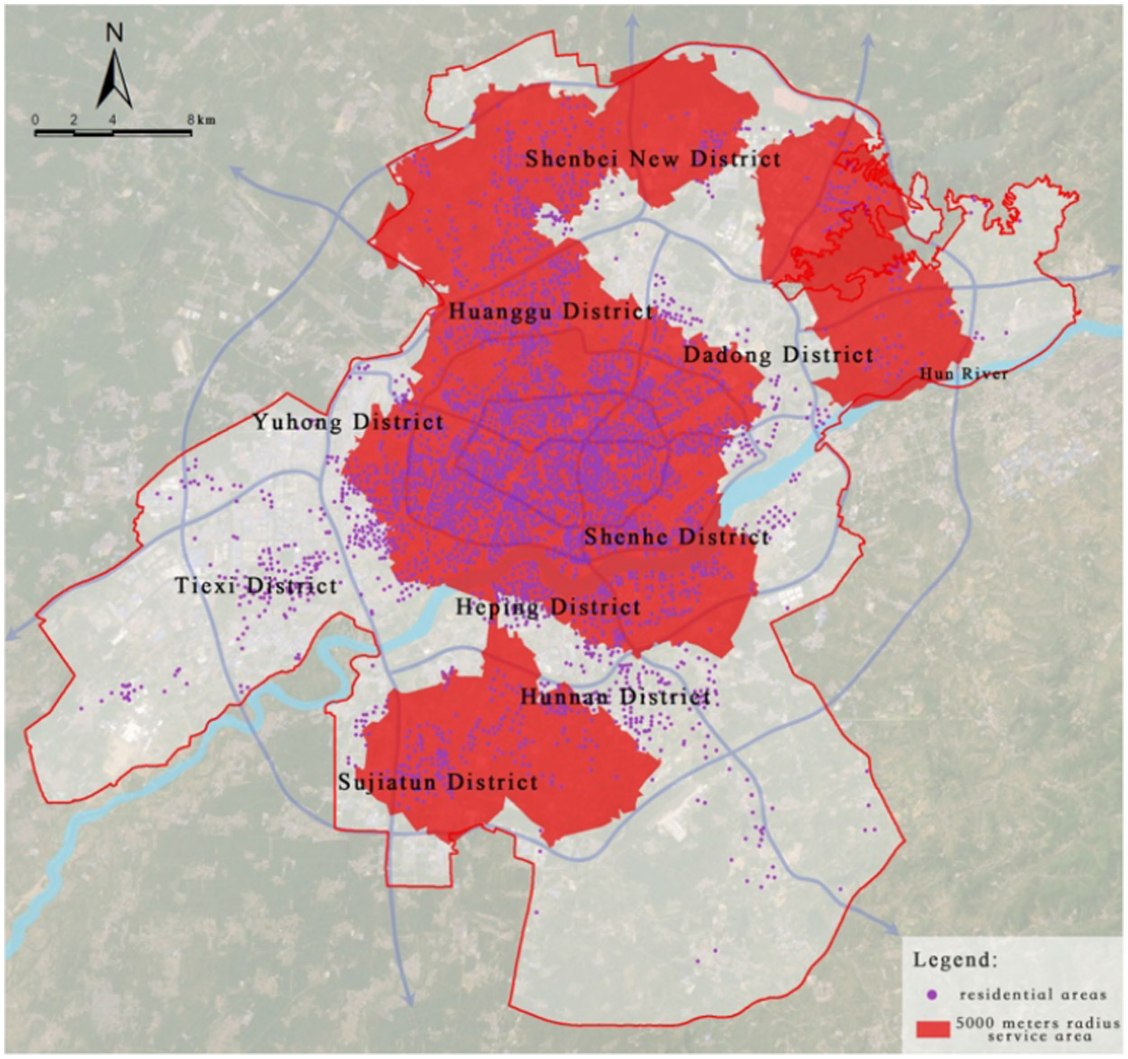
Service area analysis of city-level medical facilities.

By overlaying the healthcare facility locations with road network data and using the service area network analysis method in ArcGIS, it was found that 3,748 residential areas are within the 5,000-meter service radius, accounting for 85.05% of the total number of residential areas. The remaining 659 residential areas, which fall outside the service radius, represent 14.95% of the total (Figure 7).

**Figure 8:**
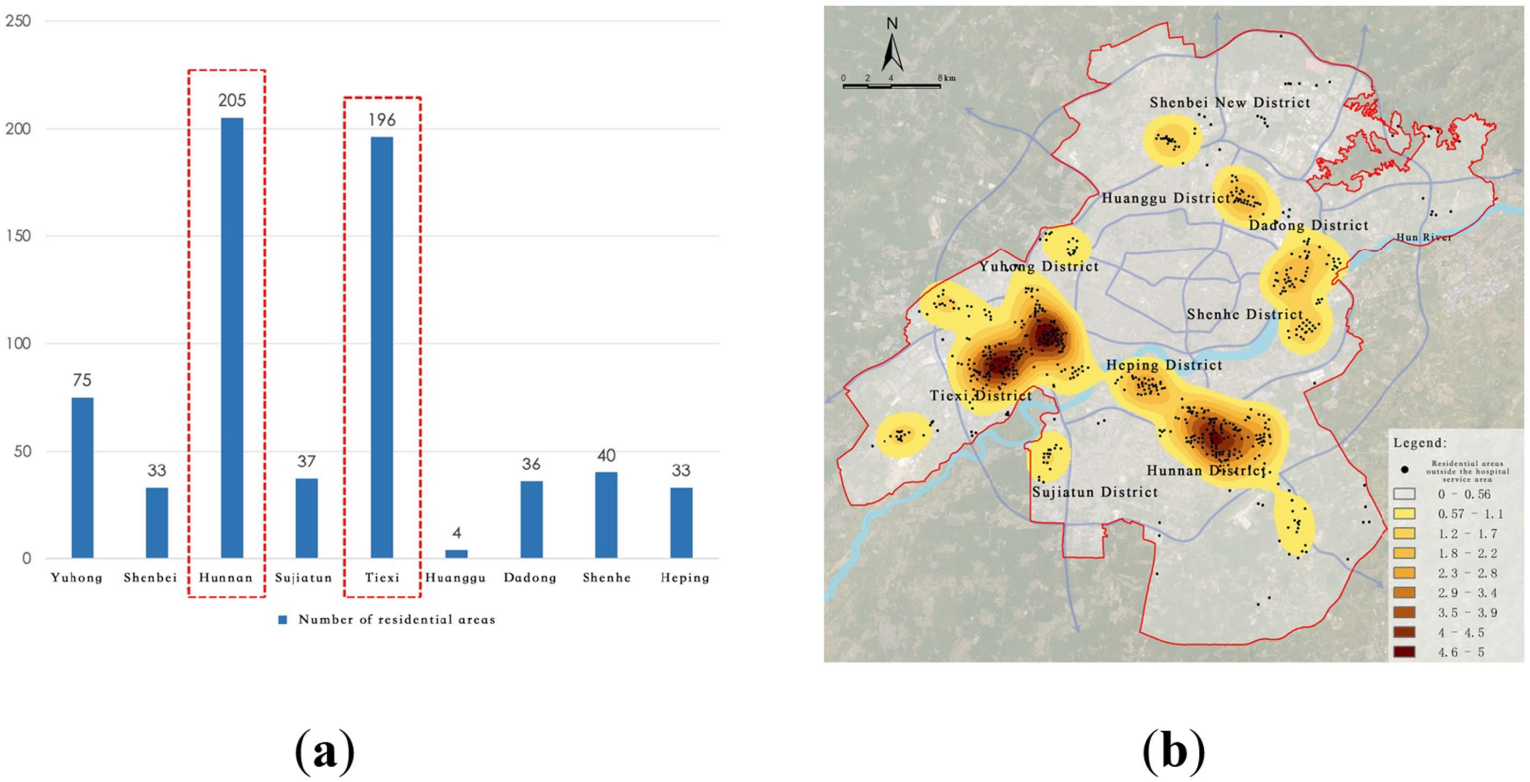
Service blind zones of city-level medical facilities. **(a)** Number of residential areas in blind zones; **(b)** Identification of city-level service blind zones.

Further analysis of these residential areas outside the service radius through kernel density analysis revealed two significant “healthcare deficiency” areas in Hunnan District and Tiexi District, highlighting a notable scarcity of healthcare facilities in these regions. Specifically, there are 205 residential areas in Hunnan District and 196 in Tiexi District that fall outside the service radius of tertiary hospitals, accounting for 60.85% of the areas not covered by the service radius. This result indicates that the peripheral regions of the city, especially emerging urban areas such as Hunnan District, still have significant gaps in healthcare facility coverage and urgently need additional high-level medical resources (Figure 8).

#### 3.2.2 “Healthcare deficiency” areas at the district level

District-level medical facilities are primarily composed of primary and secondary hospitals, serving the surrounding areas and residents within their jurisdiction. Based on the service range of these facilities, this study sets the service radius of primary hospitals at 1,500 meters and that of secondary hospitals at 3,000 meters.

**Figure 9:**
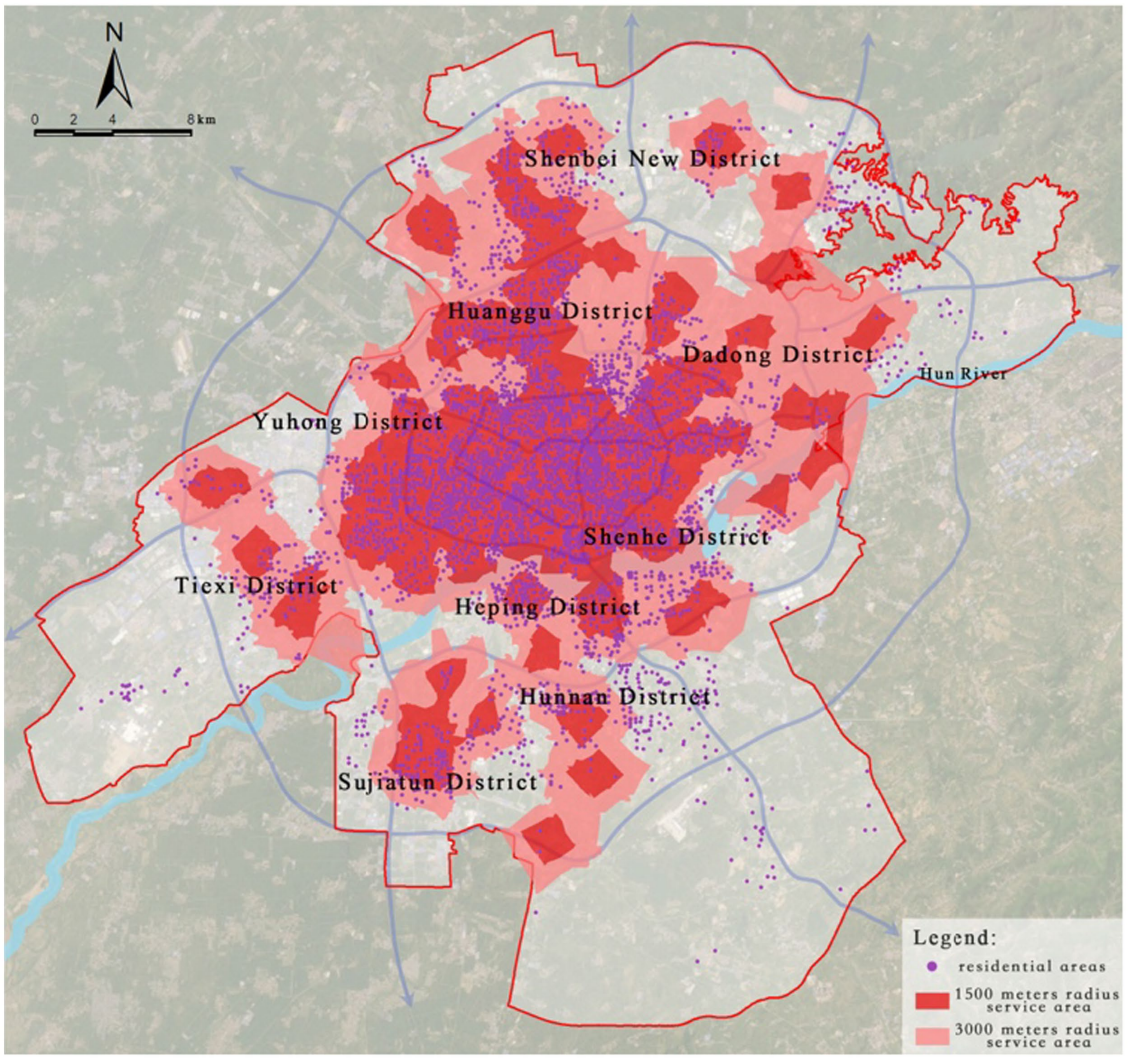
Service area analysis of district-level medical facilities.

According to the service area network analysis, 4,045 residential areas are within the respective service radii, accounting for 91.79% of the total number of residential areas, while 362 residential areas lie outside the service radii, making up 8.21% (Figure 9).

**Figure 10:**
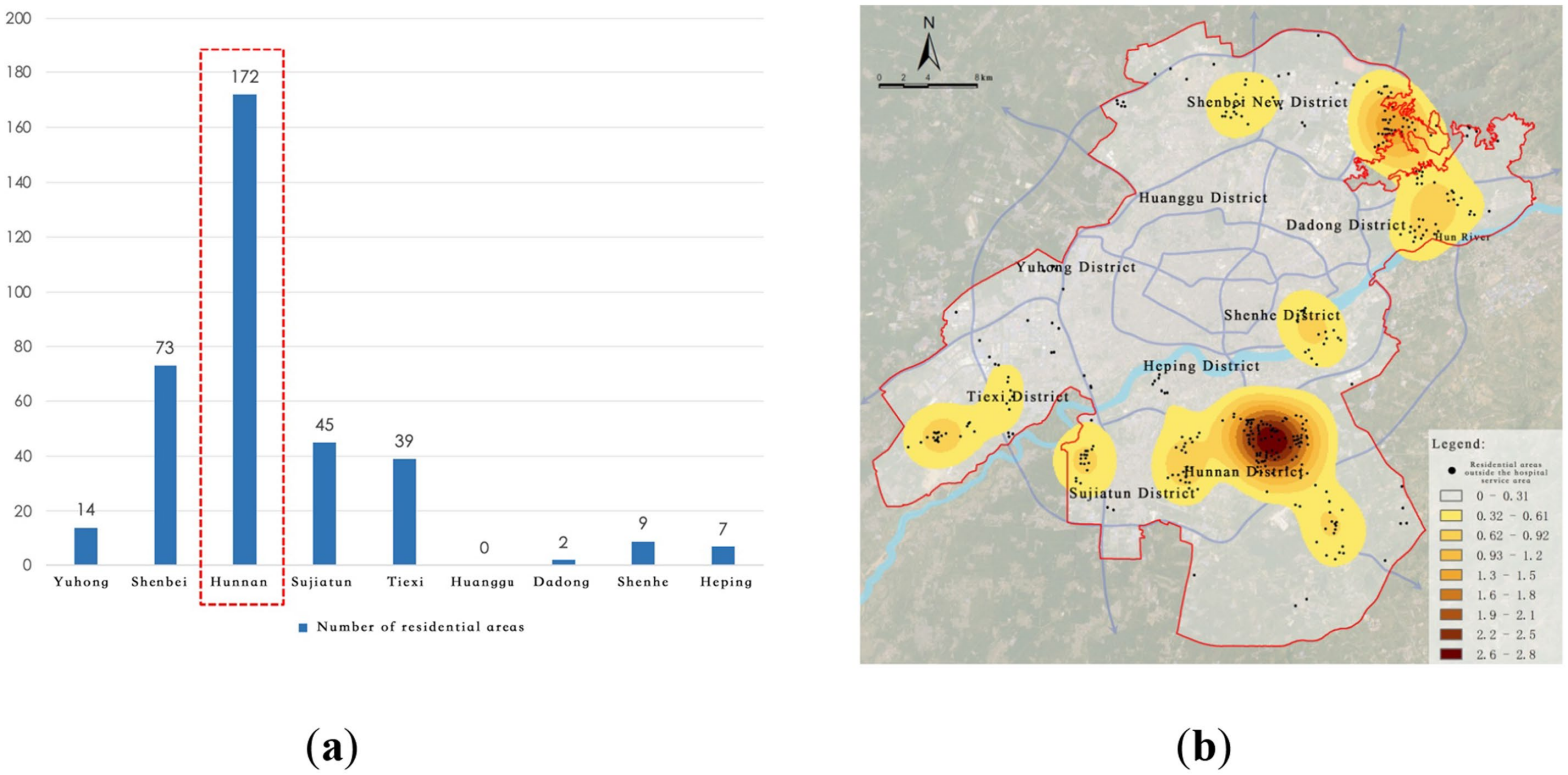
Service blind zones of district-level medical facilities. **(a)** Number of residential areas in blind zones; **(b)** Identification of district-level service blind zones.

Further extraction and kernel density analysis of the residential areas outside the service radii of primary and secondary hospitals revealed that peripheral regions such as Shenbei New District, Sujiatun District, Tiexi District, and Hunnan District generally suffer from fragmented healthcare service gaps. Particularly in the Hunnan New City High-Tech Industrial Park, 172 residential areas are not covered within the service range of primary and secondary hospitals, indicating a lack of healthcare facilities in this area, which severely impacts residents’ access to basic healthcare services (Figure 10).

#### 3.2.3 “Healthcare deficiency” areas at the community level

Community-level medical facilities are composed of community health service stations, which are responsible for providing primary healthcare services. According to the “Urban Residential Area Planning and Design Standards (GB 50180–2018)” (hereinafter referred to as the “Residential Area Standards”), this study sets the service radii of community health service stations at 300 meters, 500 meters, and 1,000 meters.

**Figure 11:**
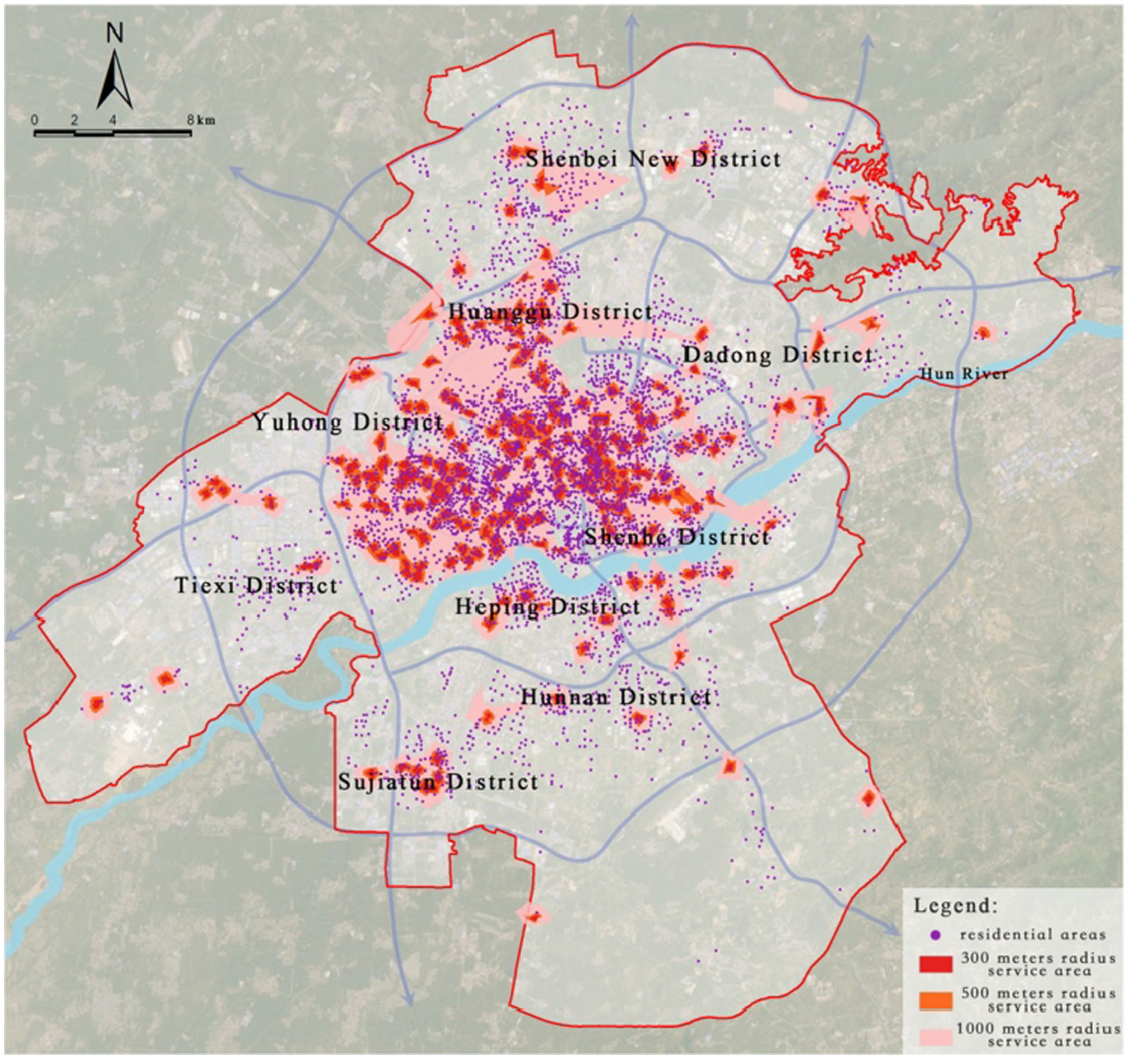
Service area analysis of community-level medical facilities.

Through road network analysis, it was found that 2,974 residential areas fall within the service range of existing community health service stations, accounting for 67.48% of the total number of residential areas, while 1,433 residential areas do not meet the requirements for public health facility construction specified in the “Residential Area Standards,” accounting for 32.52% (Figure 11).

**Figure 12:**
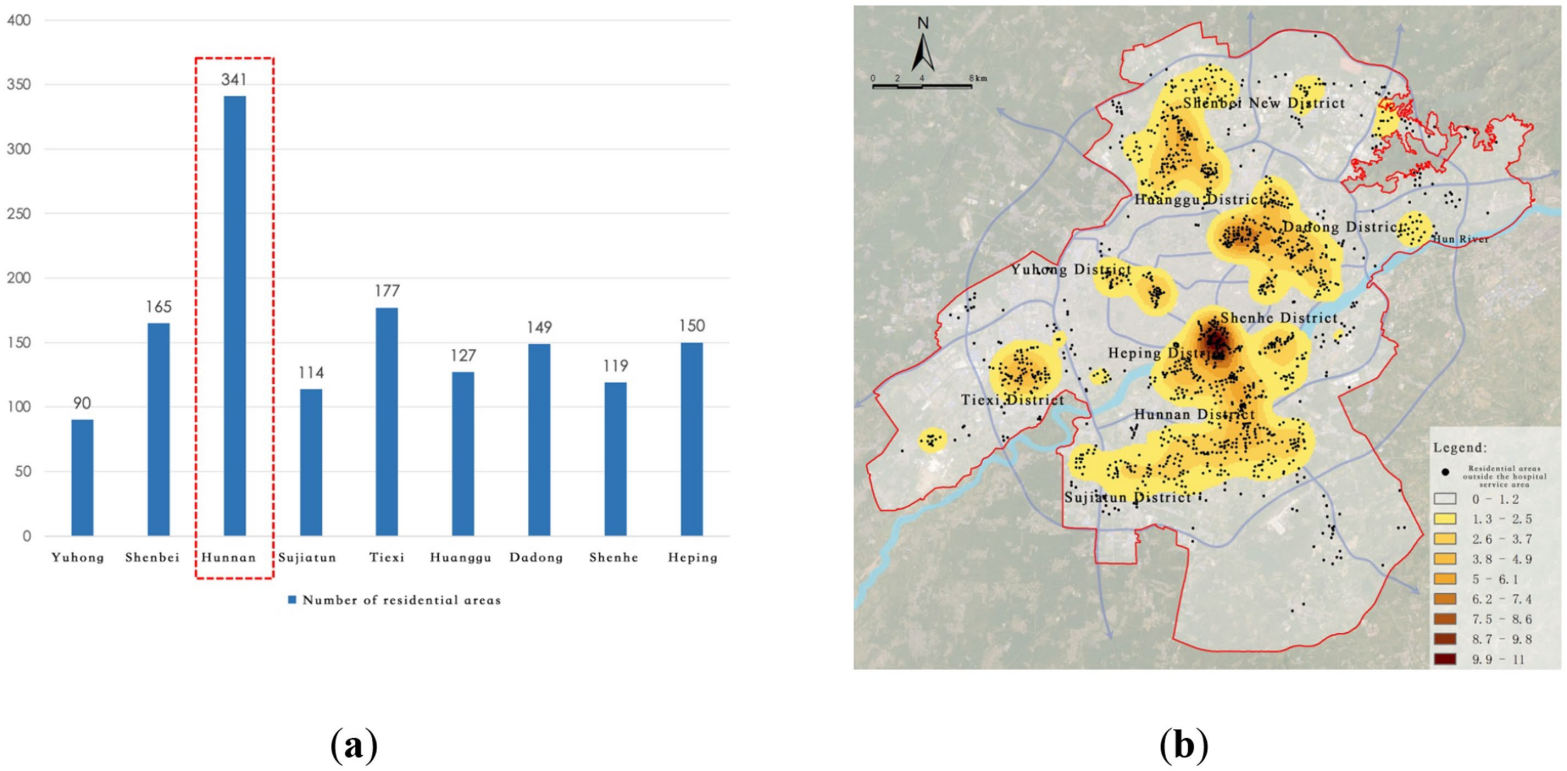
Service blind zones of community-level medical facilities. **(a)** Number of residential areas in blind zones; **(b)** Identification of community-level service blind zones.

Further extraction and kernel density analysis of the residential areas not meeting the requirements for public health facilities revealed that the highest-density “healthcare deficiency” area is located near the city library, with multiple regions such as Dadong District, Huanggu District, Shenbei New District, and Hunnan District showing fragmented “healthcare deficiency” distributions. In particular, Hunnan District has the highest number of residential areas not meeting the “Residential Area Standards,” totaling 341, indicating a severe shortage of grassroots medical resources in Hunnan, which urgently needs improvement (Figure 12).

### 3.3 Optimization of tertiary medical facility layout based on the LA model

To achieve scientific allocation of healthcare resources and comprehensive improvement of service accessibility, this study employs the Location Allocation Model (LA Model) based on the maximum coverage approach. The goal is to optimize the spatial layout of medical facilities at the city-wide, district, and community levels with a focus on “minimum facility increment + maximum service coverage.” The model optimizes the location selection of facilities by setting key parameters such as service radius and population capacity, ultimately creating a healthcare facility spatial layout that aligns with the urban population distribution characteristics and development needs.

Referring to the “Shenyang Regional Health Plan (2021–2025)” and the “14th Five-Year Plan for Health and Wellness Development in Shenyang,” by the end of 2020, Shenyang had 8.04 beds per thousand people, which is higher than cities like Guangzhou (6.38), Ningbo (6.42), and Qingdao (6.17). The planning goal is to control the number of beds to 7.6 per thousand people by 2025, emphasizing the rational control of hospital sizes and promoting the “one hospital, multiple zones” development model, while also restricting the unreasonable expansion of public hospitals.

Based on the current average number of beds and service capacities for various levels of medical institutions, the service population capacity thresholds for each level of facility in the model are set as follows (Table 2).

**Table 2:** Service standards for different levels of medical facilities.

Medical facility level	Service population capacity	Service radius setting
City-wide level	260,000 people	5,000 meters
District level	67,000 people	3,000 meters
Community level	15,000 people	1,000 meters

#### 3.3.1 Optimization of city-level medical facility layout

Considering that most of the existing tertiary hospitals are located in the core urban areas, where the surrounding medical industry system is well-established, and given the high relocation costs and policy constraints, this study adopts a strategy of minimal incremental optimization without changing existing facilities. The objective is to maximize the service coverage while ensuring that the service capacity thresholds are not exceeded, and to minimize the number of newly added facilities.

**Figure 13:**
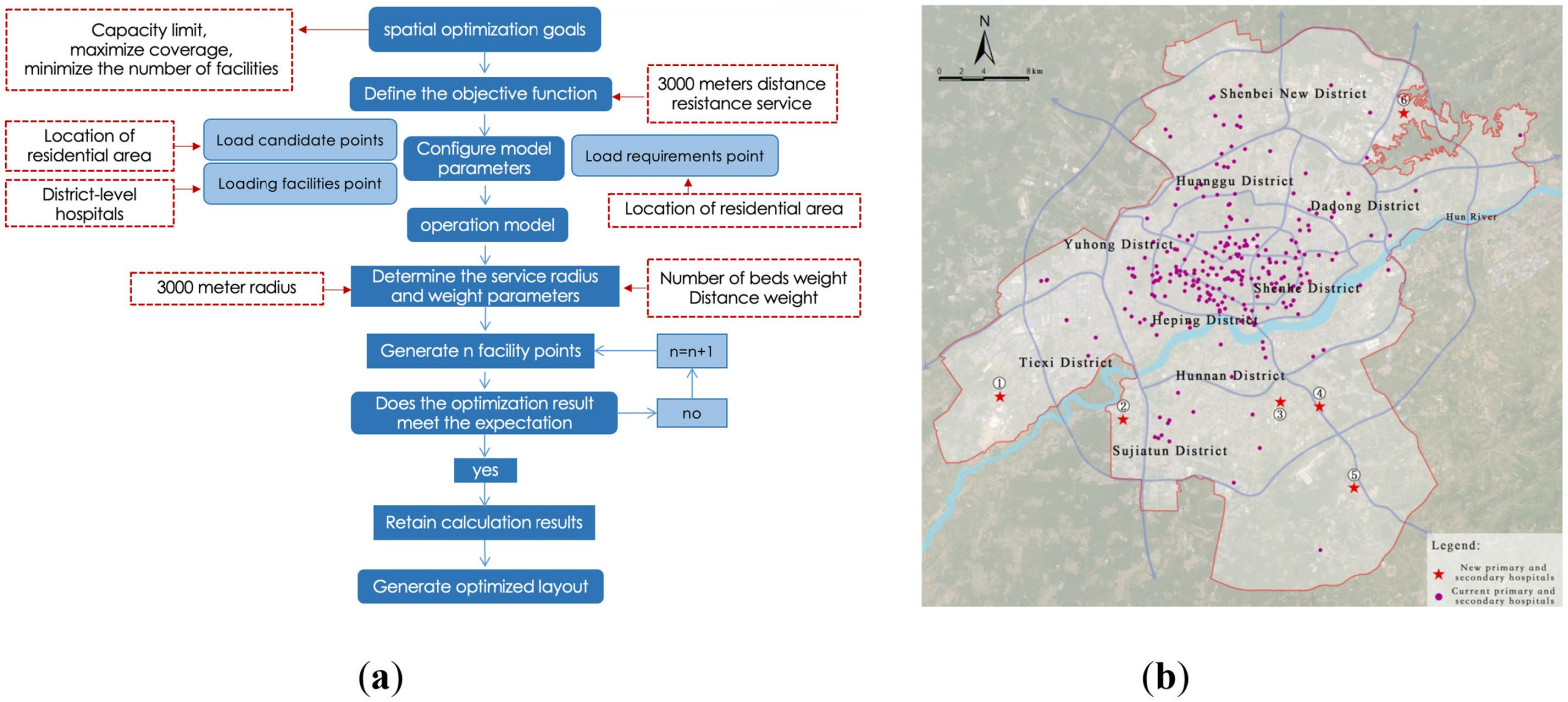
Optimization of city-level medical facility layout. **(a)** Location-allocation model results (city-level); **(b)** Proposed layout of city-level medical facilities.

Using the residential areas that are currently not covered by services as candidate sites, spatial calculations are performed based on the Location Allocation (LA) model. The results indicate that by adding six new tertiary hospitals, the service coverage of residential areas can be increased from 85.05 to 95.41%, covering a total of 4,207 residential areas (Figure 13; Table 3). Under ideal traffic conditions, the average driving time from each residential area to a tertiary hospital is approximately 5.6 min, significantly improving the accessibility of high-level medical resources in peripheral areas.

**Table 3:** Details of newly proposed city-level medical facilities.

New facility code	New facility location	District	Number of served residential areas (count)	Total number of households (households)
Point 1	Fulong Yaju	Tiexi District	104	110,927
Point 2	Wanli Yufu	Tiexi District	100	116,434
Point 3	Vanke Emerald Seasons	Hunnan District	75	98,055
Point 4	Yisheng Yushanfu	Hunnan District	97	115,237
Point 5	Poly Dakui Diwan	Shenhe District	57	73,049
Point 6	CIFI Dongyuecheng	Dadong District	55	65,943

#### 3.3.2 Optimization of district-level medical facility layout

District-level medical facilities provide services that are secondary to those of tertiary hospitals, mainly serving local residential areas. The planning of these facilities should emphasize balanced distribution and convenient access.

**Figure 14:**
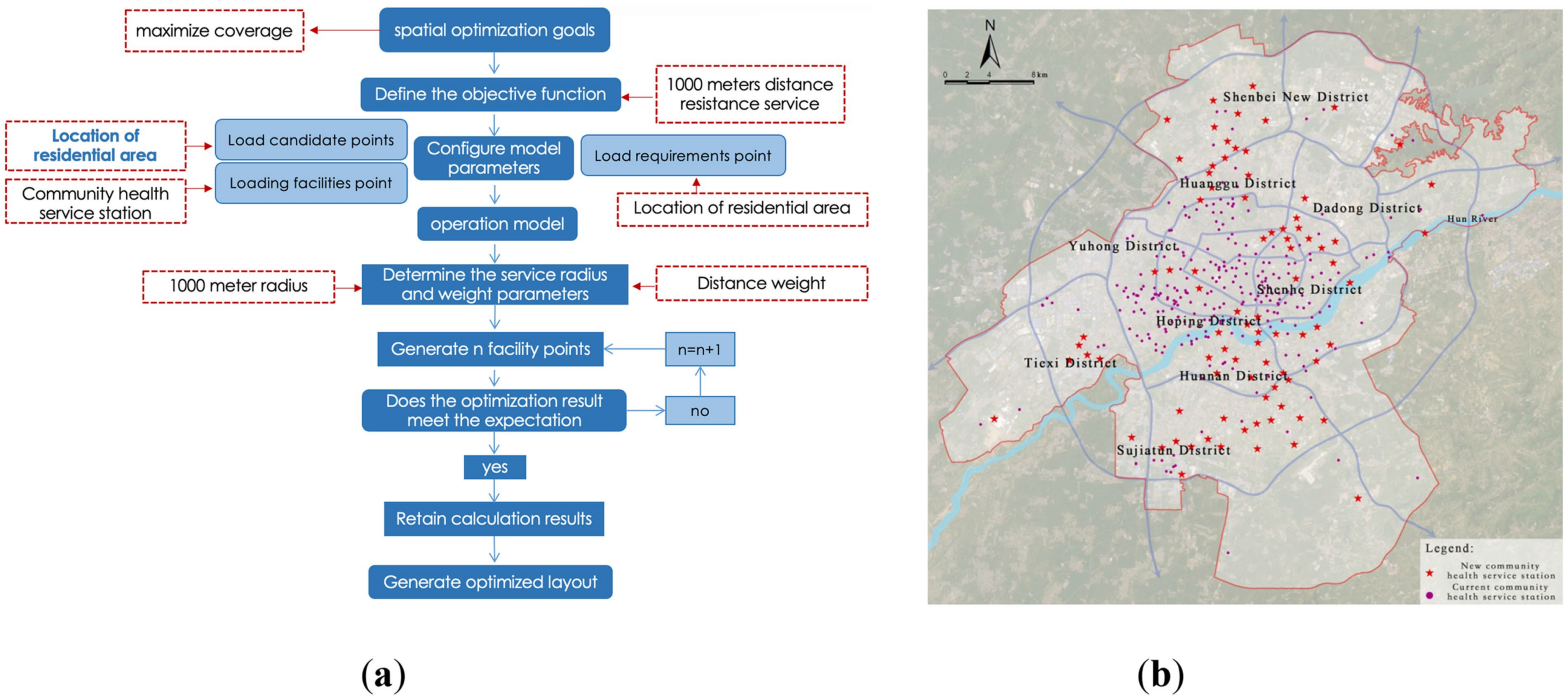
Optimization of district-level medical facility layout. **(a)** Location-allocation model results (district-level); **(b)** Proposed layout of district-level medical facilities.

This section adopts the same optimization logic and model settings as used for the city-level optimization, using current service gaps as candidate locations for the minimal incremental optimization of district-level facilities. The service radius is set at 3,000 meters, and the service population threshold is 67,000. The model results show that after adding 6 district-level hospitals, the service coverage rate increases from 91.79 to 95.55%, covering a total of 4,211 residential areas (Figure 14; Table 4). The average travel time decreases to 3.1 min. Although the increase in coverage rate is limited, the optimization effectively addresses service gaps in some peripheral areas.

**Table 4:** Details of newly proposed district-level medical facilities.

New facility code	New facility location	District	Number of served residential areas (count)	Total number of households (households)
Point 1	Hongze International	Tiexi District	19	18,326
Point 2	Huaxi Town	Sujiatun District	16	20,204
Point 3	Longhu Yunfeng Yuan	Hunnan District	24	27,429
Point 4	Tingyu Guanlan	Hunnan District	26	27,870
Point 5	Shengli Jiayuan	Sujiatun District	16	15,499
Point 6	Xiangfeng Mountain Water International	Shenbei New District	33	27,314

#### 3.3.3 Optimization of community-level medical facility layout

Community-level facilities (i.e., community health service stations) form the foundation of the medical service system, and their rational layout is crucial for establishing a “15-min health living circle.” Based on the evaluation of the current layout, the service coverage rate is only 67.48%, indicating significant room for improvement. This section proposes two optimization strategies: Incremental Optimization Plan 1 and Spatial Redistribution Plan 2, to compare and evaluate the feasibility and advantages of the community-level facility layout.

**Figure 15:**
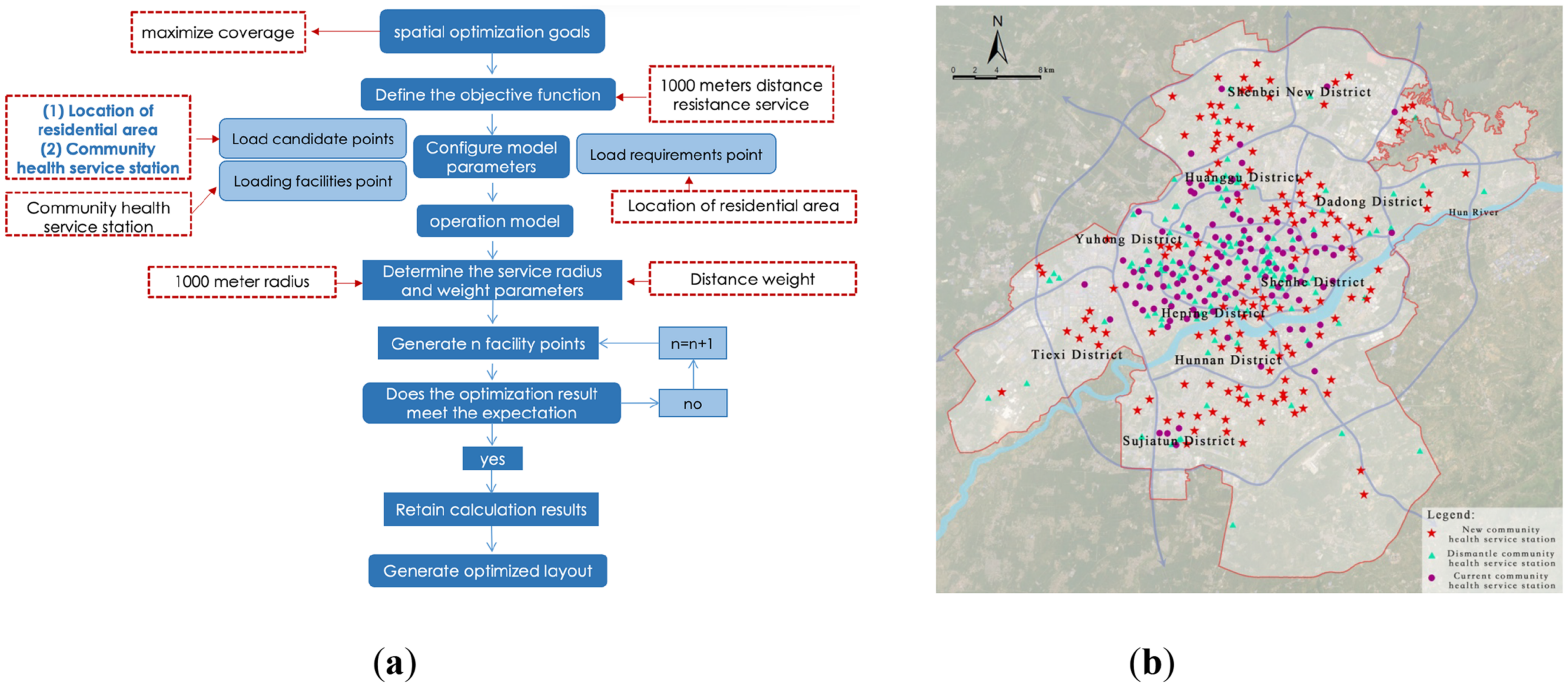
Optimization of community-level medical facility layout. **(a)** Location-allocation model results (community-level) of plan 1; **(b)** Proposed layout of community-level medical facilities of plan 1.

(1) Plan 1: Facility Incremental Optimization Path:

This strategy retains the current layout of community health service stations, selecting residential areas not within the 1,000-meter service radius as candidates for new facilities, with the goal of maximizing service coverage. Model calculations indicate that by adding 84 new facilities, the service coverage rate increases to 86.63% (Figure 15). However, observation reveals that in some central urban areas, the distribution of facilities is dense, leading to redundancy in service capacity, suggesting that simple expansion could result in resource wastage.

**Figure 16:**
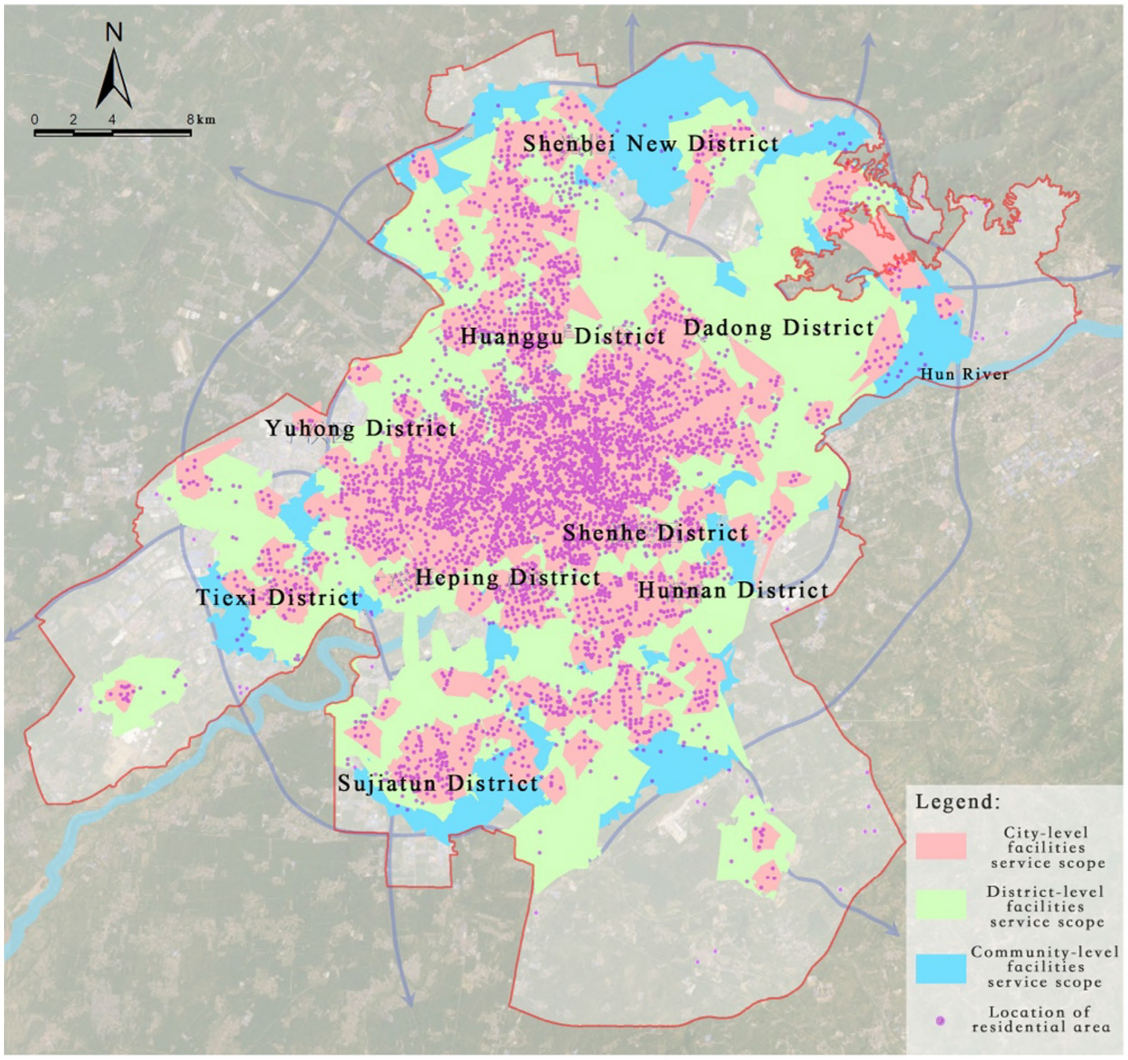
Optimization of community-level medical facility layout. **(a)** Location-allocation model results (community-level) of plan 2; **(b)** Proposed layout of community-level medical facilities of plan 2.

(2) Plan 2: Facility Redistribution Optimization Path:

Considering that the construction and relocation costs of community health service stations are relatively low, Plan 2 attempts to optimize the overall resource allocation through spatial redistribution, using existing facilities and residential service gaps as candidate points. The results show that by adjusting the layout without increasing the total number of facilities, the service coverage rate can be improved to 88.59% (Figure 16). This strategy is more cost-efficient in terms of land use, personnel management costs, and equitable access for residents compared to the simple expansion model.

A comparison of the two plans (Table 5) demonstrates that Plan 2, with its spatial redistribution logic, offers superior cost-effectiveness and service equity, confirming the principle that “facility redistribution is superior to blind expansion” for optimizing grassroots medical layout.

**Table 5:** Comparison of optimization plans for community health service stations.

Comparison indicator	Plan 1	Plan 2
Total After Optimization	350 units	260 units
Number of New Units	84 units	−6 units
Number of Relocations	—	143 units
Service Area Coverage After Optimization	86.63%	88.59%
Service Area Coverage Increase Rate	19.15%	21.11%

#### 3.3.4 Evaluation of optimization effectiveness and result validation

To comprehensively evaluate the practical effectiveness of the LA model optimization results, this study validates the results by comparing the changes in service area coverage. After optimization, all levels of medical facilities show significant improvements in service coverage (Table 6). Specifically, the coverage rate for city-level facilities increases from 85.05 to 95.41%, a growth of 10.36%; for district-level facilities, the coverage rate increases from 91.79 to 95.55%, a growth of 3.76%; and for community-level facilities, the coverage rate shows the most significant improvement, rising from 67.48 to 88.59%, a growth of 21.11%.

**Table 6:** Validation statistics of the optimized layout for tertiary medical facilities.

Medical facility level	Original service area coverage (%)	Optimized service area coverage (%)	Coverage improvement (%)
Tertiary Hospitals	85.05	95.41	10.36
Primary and Secondary Hospitals	91.79	95.55	3.76
Community Health Service Stations	67.48	88.59	21.11

**Figure 17:**
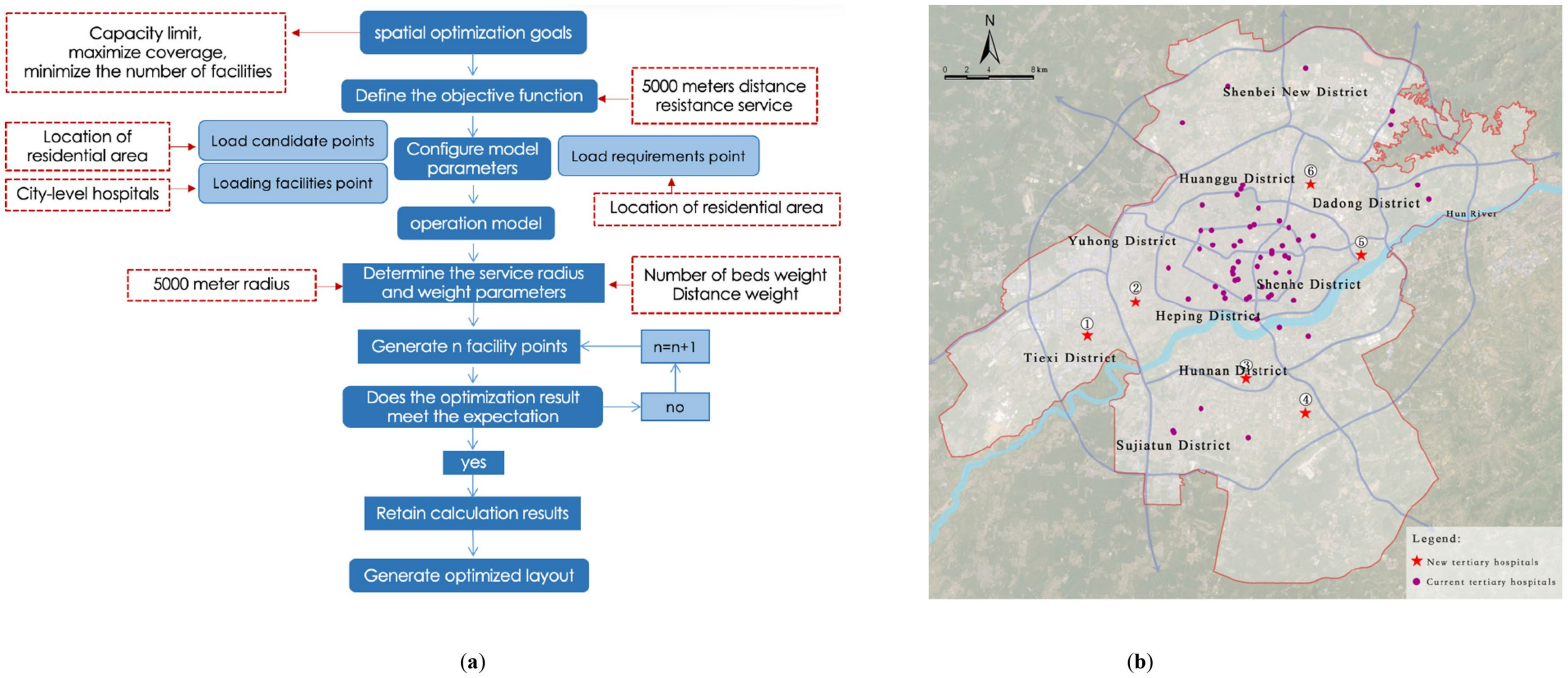
Validation of service coverage for all three levels of medical facilities.

Overall, after optimization, the combined service coverage rate of all levels of medical facilities reaches 98.98%, achieving a significant improvement in spatial equity and resource efficiency. It is especially noteworthy that district-level facilities already had a relatively high coverage rate before optimization (Figure 17), and the service blind spots were distributed in a discrete manner, meaning the impact of adding new facilities on coverage improvement is limited. Future optimization efforts should focus more on the spatial redistribution of community-level service facilities and the appropriate addition of high-capacity city-level medical institutions in peripheral new districts, to create a more resilient and inclusive medical facility spatial network.

## 4 Discussion

### 4.1 Key research findings

This study systematically reveals the spatial distribution characteristics and supply–demand matching efficiency of medical facility layouts in Shenyang using multi-source data and spatial analysis models. The main findings of the study are as follows:

(1) “Centralized Concentration–Peripheral Scarcity” Spatial Distribution Pattern.

The medical facilities in Shenyang show a significant spatial imbalance, manifested as a “centralized concentration - peripheral scarcity” gradient distribution. Specifically, tertiary hospitals and primary and secondary hospitals are primarily concentrated in the old city area north of Hunhe River, especially in multi-core areas such as Tiexi Square and the First Affiliated Hospital of China Medical University, forming high-density medical resource concentration hubs. However, in emerging development areas like Hunnan New City and Shenbei New District, the coverage of medical facilities is significantly insufficient, resulting in a severe gap in medical resources. The spatial layout of community health service stations, in particular, misaligns with population density distribution, especially in the population-concentrated areas of Hunnan New City. Multiple newly built residential communities have failed to provide basic medical facilities according to the “Residential District Standards,” leading to a severe shortage of grassroots medical services in this area. This spatial inequality is further confirmed by the calculated Gini coefficient of 0.523 for the distribution of medical beds across all sub-districts in the central urban area, indicating a high degree of imbalance. Some districts, such as Sujiatun (0.68) and Hunnan (0.62), show extreme disparities, where large portions of the population reside in areas with no available beds, while a few urban centers hold a disproportionately high share of medical resources.

(2) Hierarchical Differences in Medical Resource Supply–Demand Matching Efficiency.

Through analysis of road network service areas, this study found that the supply–demand matching efficiency of medical resources in Shenyang shows significant hierarchical differences. Although the service coverage of tertiary hospitals in the city’s core areas reaches 85.05%, residents in peripheral areas, particularly those outside the service radius, face a significant increase in time and spatial costs when accessing quality medical resources. Meanwhile, the service blind spots of primary and secondary hospitals account for 8.21%, mainly concentrated in Hunnan New City’s high-tech industrial park and newly developed residential areas. These areas lack adequate medical service facilities, forcing residents to bear high time and transportation costs when seeking medical care. As an essential component of the grassroots medical system, the service blind spots of community health service stations largely overlap with densely populated areas. In particular, in Hunnan District, many newly built communities failed to meet the medical facility construction requirements of the “Residential District Standards,” further exacerbating the mismatch between grassroots medical service supply and demand. This tiered inefficiency corresponds with the overall inequality identified in the Gini analysis, highlighting that not only are resources centrally concentrated, but their misalignment across hierarchical levels amplifies the accessibility gap for marginalized urban populations.

(3) Verification of the Effectiveness of the Incremental and Stock Coordinated Regulation in the Spatial Optimization Model.

The spatial layout optimization simulations performed using the LA model in this study verified the effectiveness of incremental and stock coordinated regulation. Specifically, by optimizing the configuration and adding six tertiary hospitals and six primary and secondary hospitals, the service coverage rates were increased to 95.41 and 95.55%, respectively, significantly improving medical service accessibility in peripheral areas. For the optimization of community health service stations, the stock reallocation plan proposed in this study, compared to the pure incremental approach of adding new facilities, effectively saves land resources and increases the coverage rate to 88.59%. This result validates the optimization logic of “space reallocation is superior to facility expansion,” providing strong theoretical and practical guidance for the rational layout of grassroots medical facilities in Shenyang.

### 4.2 Layout optimization strategies

This study identifies key challenges in the spatial configuration of healthcare facilities in Shenyang, including the structural imbalance of “central concentration–peripheral scarcity,” disparities in supply–demand matching efficiency across facility levels, and the underutilization of existing resources. The results, including a Gini coefficient of 0.523 at the city scale and even higher values in peripheral districts such as Sujiatun and Hunnan, provide quantitative evidence of significant spatial inequality in medical resource allocation. In response, this section proposes a set of hierarchically differentiated and operable spatial optimization strategies, aimed at enhancing both equity and systemic efficiency:

(1) Population-Oriented “Hierarchical Compensation” to Address Peripheral Service Gaps.

To mitigate the overconcentration of high-level resources in core districts and the lack of medical services in newly developed urban areas, a population- and accessibility-constrained allocation framework is proposed: Tertiary hospitals should retain high-level diagnostic and treatment capabilities in the core urban area, while selectively relocating or expanding redundant capacity into underserved zones such as Hunnan High-Tech Zone and Shenbei New District to improve regional accessibility; Primary and secondary hospitals should be incrementally deployed along major transit corridors, within high-density residential areas and integrated work–residence new towns. Facility siting should be guided by network-based accessibility models and population growth projections; Community-level facilities, especially health service stations, should be optimized based on 15-min neighborhood standards (55), incorporating population density and identified service blind spots to target under-served blocks with either new facilities or strategic consolidation, thereby strengthening grassroots healthcare access.

(2) “Relocate–Adjust–Merge”: A Dynamic Consolidation Mechanism for Community-Level Facilities.

To address overlapping service areas, underutilization, and spatial mismatches between facility capacity and population needs, a dynamic restructuring strategy for stock-based optimization is proposed: Leverage GIS-based redundancy detection models and spatial coverage simulations to identify low-efficiency sites for merging, functional integration, or geographic repositioning; Promote the co-location of community health centers with older adult care, rehabilitation, and mental health services to form multi-functional health nodes at the community scale (56); Establish a mechanism for periodic facility review and flexible adjustment, enabling a rolling configuration model that adapts to urban demographic shifts and redevelopment cycles.

(3) Institutional Embedding of Tiered Diagnosis and Two-Way Referral to Improve Systemic Coordination.

Optimizing spatial layout must be synchronized with institutional arrangements. In light of the evidence on efficiency differentials across medical levels, the following is recommended: Couple patient pathway simulations with spatial planning models to assess the impact of varying referral rates on system performance; Drawing on Beijing’s experience, use insurance reimbursement policies to encourage first contact at the grassroots level, followed by upward referral when necessary, supporting a stratified and orderly healthcare delivery model (57); Embed functional role definition and referral logic into the planning stage, fostering spatially linked networksacross neighborhood and regional levels to relieve pressure on top-tier hospitals and enhance responsiveness across the system.

(4) Smart Integration and Behavioral Responsiveness to Support Flexible and Precision-Oriented Facility Planning.

Given the dynamic evolution of urban spatial structures and the increasing complexity of patient behaviors, smart technologies should be harnessed to support adaptive planning: Develop integrated multi-source data platforms that combine healthcare operational data, spatiotemporal population distribution, and travel behaviors to dynamically detect service overloads and spatial blind spots (58); Use mobile phone trajectory and OD data to analyze weekday–weekend and day–night variation in demand, adjusting service boundaries and operational strategies accordingly; Encourage tertiary hospital physicians to rotate into primary facilities and promote telemedicine platforms, particularly in edge zones and for vulnerable populations, building a hybrid digital-physical support system to expand the effective reach of grassroots care.

## 5 Limitations and future outlook

This study systematically analyzes the spatial distribution and optimization strategies of multi-tiered healthcare facilities in Shenyang using multi-source data and spatial modeling approaches. While the research offers methodological innovations and empirical insights, several limitations remain, which, rather than undermining the core findings, highlight potential directions for future research.

First, limitations in data completeness and the heterogeneity of individual behavior warrant further improvement. The POI and AOI data used in this study were primarily derived from Baidu Maps (2024 edition). Although these datasets are relatively up to date, their static nature may not fully reflect the dynamic evolution of urban development. Moreover, the service area analysis relies on static road networks and uniform service radii, which do not account for differences in travel behavior by age (59), mobility preferences (60), or socioeconomic status (61). Future research could incorporate individual-level mobility data (e.g., GPS or mobile phone signaling) to enhance behavioral realism in modeling healthcare service coverage.

Second, the transferability of the methodological framework needs validation across diverse urban contexts. While Shenyang serves as a representative case of an aging industrial city, urban systems vary widely in spatial structure, development stage, and healthcare governance models. To enhance generalizability, future studies could apply this framework to a wider range of cities—including rapidly developing urban regions and peri-urban areas—conducting cross-case comparisons to extract more broadly applicable insights for healthcare spatial planning.

Third, the assumptions around service radii require further refinement through sensitivity and elasticity analysis. This study adopts service radius thresholds (5 km for tertiary, 3 km for secondary, and 1 km for primary facilities) based on national planning standards. While these parameters account to some extent for the mobility of older adult populations, they may oversimplify real-world dynamics. Accessibility is also influenced by transportation modes, road network complexity, disease urgency, and user perceptions (62, 63). Future work should incorporate time–cost thresholds and impedance-based sensitivity analysis, potentially using big data regression models to derive empirically grounded service areas based on actual patient behavior.

Fourth, the study does not fully address multidimensional accessibility or the role of digital health tools. The current analysis emphasizes spatial accessibility but does not comprehensively incorporate non-spatial factors such as health literacy, socioeconomic status, insurance coverage, and cultural-linguistic barriers (64). Future research should adopt a multidimensional framework that integrates spatial, social, and institutional dimensions of access. Moreover, digital health tools such as telemedicine platforms and smart health apps should be examined for their potential to enhance access for vulnerable populations and improve the adaptability of the healthcare delivery system.

Fifth, the role of developers and citizen participation in public service provision merits closer attention. In China’s urban development practice, developers have become important actors in the provision of public facilities. Through mechanisms such as floor area ratio bonuses, mixed-use zoning incentives, and delegated facility construction, developers contribute significantly to the planning and delivery of healthcare and other public services. Successful examples include Shenzhen’s “Community Health Renaissance” initiative and Shanghai’s “15-Minute Living Circle” guidelines (65, 66). Future research should explore the interactions between planning policies and developer behavior, as well as how market mechanisms can be aligned with equitable spatial provision. On the other hand, citizen participation in planning remains largely reactive. While mechanisms like public consultations exist, decision-making is still predominantly government-led (67). Future studies should investigate institutional mechanisms to embed user feedback (e.g., participatory mapping, digital platforms) into the early stages of healthcare facility planning.

Sixth, home care as a complementary layer in the hierarchical healthcare system deserves greater attention. Although this study focuses on the spatial configuration of tertiary, secondary, and primary healthcare institutions, home care plays a crucial role in meeting the needs of aging populations and patients with chronic conditions (68). As an extension of the primary care network, home care services can reduce pressure on hospitals and improve continuity of care. It enhances patient autonomy, reduces caregiver burden, and improves healthcare worker job satisfaction (69). Future research should explore how home care services can be spatially integrated into the existing system—through health records, remote monitoring, and neighborhood-based support—to construct a resilient and people-centered healthcare delivery model.

In summary, while this study provides empirical evidence and methodological insights for multi-tiered healthcare spatial optimization, further research is needed to refine behavioral data integration, improve model adaptability, and expand toward more inclusive, flexible, and responsive healthcare planning systems.

## 6 Conclusion

This study systematically investigated the spatial patterns, supply–demand matching efficiency, and optimization strategies of hierarchical healthcare facilities in Shenyang, utilizing multi-source data and spatial modeling techniques. The core findings and contributions of this research can be summarized as follows:

First, the spatial layout of medical facilities in Shenyang exhibits a pronounced “centralized concentration–peripheral scarcity” pattern. High-level healthcare resources are predominantly clustered in traditional urban centers north of the Hun River, particularly in Tiexi and Heping districts, while emerging development zones such as Hunnan New City and Shenbei New District face significant shortages. This spatial mismatch is especially evident in the distribution of community health service stations, where many newly developed residential communities fail to meet the facility standards defined by the “Residential District Guidelines,” leading to widespread grassroots service blind spots.

Second, the supply–demand matching efficiency of medical resources shows notable differences across facility tiers. While tertiary hospitals provide relatively high service accessibility, residents in peripheral areas experience significantly higher spatial and temporal costs to reach them. Similarly, primary and secondary hospitals, as well as community facilities, present visible blind spots in rapidly urbanizing districts. A Gini coefficient of 0.523 further confirms the inequality in the spatial distribution of beds relative to population across districts, with some areas showing severe oversupply or undersupply, indicating an urgent need for spatial restructuring.

Third, this study demonstrates the effectiveness of a spatial optimization strategy based on the Location-Allocation (LA) model, which integrates both incremental additions and stock-based reallocation of medical facilities. Simulation results show that strategically adding six tertiary and six primary/secondary hospitals can raise coverage rates above 95%, significantly improving accessibility in underserved areas. For community health service stations, a stock reallocation approach proves more efficient than merely increasing facility numbers, enhancing spatial equity while reducing land and resource consumption.

Building on these insights, the study proposes a four-tiered set of spatial strategies to improve equity and efficiency: (1) a population-oriented hierarchical supplementation strategy to address peripheral service gaps; (2) a dynamic consolidation mechanism for community facilities to enhance resource utilization; (3) spatial integration of tiered healthcare referral systems to improve systemic coordination; and (4) the use of smart technologies to enable adaptive facility allocation and precision service delivery. These strategies offer practical solutions to optimize Shenyang’s healthcare network while advancing the goals of equitable access and system resilience.

Moreover, regarding the replicability of the proposed framework in other urban contexts, this study acknowledges that differences in healthcare systems, urban morphology, and demographic structures may limit direct transferability. However, the methodological components—including the tiered facility identification logic, service accessibility evaluation, and spatial optimization model—possess a high degree of adaptability. Key parameters such as service radii and weighting factors can be adjusted according to local conditions, and core data inputs (e.g., facility locations, population grids, road networks) are widely accessible across cities. Future research should further validate this framework in diverse urban settings and explore scenario-specific adaptations to strengthen its generalizability and practical value.

In conclusion, this study offers both empirical evidence and methodological innovation for the spatial optimization of healthcare facilities in Shenyang. It also provides transferable insights for other cities seeking to enhance the equity, efficiency, and sustainability of urban healthcare systems in the era of digitalization and spatially informed governance.

## Data Availability

The original contributions presented in the study are included in the article/supplementary material, further inquiries can be directed to the corresponding author.
